# Biotransformation of (−)-α-Bisabolol by *Absidia coerulea*

**DOI:** 10.3390/molecules27030881

**Published:** 2022-01-27

**Authors:** Jisu Park, Fubo Han, Ik-Soo Lee

**Affiliations:** College of Pharmacy, Chonnam National University, Gwangju 61186, Korea; qkrwltn9410@naver.com (J.P.); hanfubo0306@gmail.com (F.H.)

**Keywords:** (−)-α-bisabolol, microbial transformation, *Absidia coerulea*

## Abstract

(−)-α-Bisabolol, a bioactive monocyclic sesquiterpene alcohol, has been used in pharmaceutical and cosmetic products with anti-inflammatory, antibacterial and skin-caring properties. However, the poor water solubility of (−)-α-bisabolol limits its pharmaceutical applications. It has been recognized that microbial transformation is a very useful approach to generate more polar metabolites. Fifteen microorganisms were screened for their ability to metabolize (−)-α-bisabolol in order to obtain its more polar derivatives, and the filamentous fungus *Absidia coerulea* was selected for scale-up fermentation. Seven new and four known metabolites were obtained from biotransformation of (−)-α-bisabolol (**1**), and all the metabolites exhibited higher aqueous solubility than that of the parent compound **1**. The structures of newly formed metabolites were established as (1*R*,5*R*,7*S*)- and (1*R*,5*S*,7*S*)-5-hydroxy-α-bisabolol (**2** and **3**), (1*R*,5*R*,7*S*,10*S*)-5-hydroxybisabolol oxide B (**4**), (1*R*,7*S*,10*S*)-1-hydroxybisabolol oxide B (**5**), 12-hydroxy-α-bisabolol (**7**), (1*S*,3*R*,4*S*,7*S*)- and (1*S*,3*S*,4*S*,7*S*)-3,4-dihydroxy-α-bisabolol (**8** and **10**) on the basis of spectroscopic analyses. These compounds could also be used as reference standards for the detection and identification of the metabolic products of **1** in the mammalian system.

## 1. Introduction

**Figure 1:**
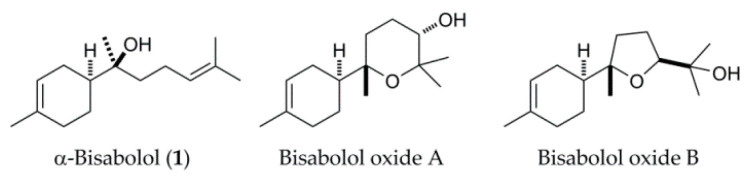
Chemical structures of α-bisabolol, bisabolol oxides A and B.

(−)-α-Bisabolol (**1**), a natural monocyclic sesquiterpene alcohol also known as levomenol, has been found in essential oils of various plants such as *Matricaria recutita* and *Alpinia officinarum* Hance [1,2]. It was reported to be nontoxic and regarded as safe by the Food and Drug Administration (FDA) and has been used in pharmaceutical and cosmetic products for its anti-inflammatory, skin soothing and moisturizing properties [3,4,5]. (−)-α-Bisabolol was shown to significantly reduce LPS-induced production of NO and PGE_2_ in a dose-dependent manner and to downregulate the expression of pro-inflammatory mediators iNOS and COX-2 via inhibition of AP-1 and NF-κB [5]. Control of the overproduction of inflammatory mediators may facilitate the treatment of inflammation linked diseases [6]. 12-*O*-Tetradecanoyl-phorbol-13-acetate (TPA)-induced ear thickness and histopathological damage in the ear tissue were significantly inhibited by (−)-α-bisabolol [6]. It exhibited significant antileishmanial activity against promastigotes with IC_50_ values of 8.07 μg/mL (24 h) and 4.26 μg/mL (48 h) during the in vitro studies and did not lead to the appearance of toxicity or side effects after the administration of (−)-α-bisabolol in healthy and infected hamsters [7,8]. The biological studies also revealed that **1** exhibited anti-irritant, antibacterial, antinociceptive, gastroprotective, cardioprotective and anticancer properties [4,9,10,11,12,13].

(−)-α-Bisabolol shows a tendency to be oxidized, with bisabolol oxides A and B being major oxidation products (Figure 1) [4]. α-Bisabolol oxide A has been reported to show higher antioxidant activity than α-bisabolol, with IC_50_ at 1.50 mg/mL and 43.88 mg/mL for DPPH radical scavenging ability, respectively [14]. The bisabolol oxide-rich matricaria oil (25.5% α-bisabolol oxide B and 21.5% α-bisabolol oxide A) was reported to exhibit antihyperalgesic and antiedematous effects [15]. Bisabolol oxide showed higher antibacterial activity than α-bisabolol during the studies against gram-positive and gram-negative bacteria [16]. Miyazawa and colleagues have reported that bisabolol oxide B was transformed from **1** by *Aspergillus niger* and *Glomerella cingulata*, and then further converted into its hydroxylated derivatives [17]. More recently, *Bipolaris sorokiniana* and *Thamnidium elegans* were applied as the biocatalysts to produce bisabolol oxide B from **1** [18,19].

**Figure 2:**
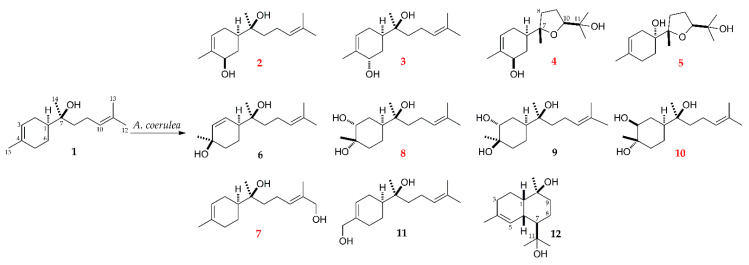
Chemical structures of (−)-α-bisabolol (**1**) and its metabolites (**2**–**11**).

Microbial transformation is a well-established green technique with high selectivity and efficiency to produce new and unique derivatives of bioactive substrates which are difficult to acquire by chemical synthesis under mild conditions [20,21]. It has been reported that microbial transformation of sesquiterpenes can result in derivatives with enhanced biological activities [22,23]. For example, 13-hydroxynootkatone exhibited stronger cytotoxic activity than its parent compound nootkatone against the lung cancer cell line A549 (IC_50_: 36 μg/mL and 58.4 μg/mL, respectively) [24], and 14-hydroxymethyl caryophyllene oxide showed more potent inhibitory activity against the enzyme butyryl cholinesterase than its parent compound caryophyllene oxide (IC_50_: 10.9 μM and 208.4 μM, respectively) (Supplementary Figure S1) [22]. Furthermore, microbial transformation is highly useful in producing more polar metabolites, leading to improved aqueous solubility [25,26]. In addition, microbial transformation has been regarded as an effective and useful experimental method to mimic and predict the mammalian metabolism of pharmaceutical agents [21,27].

(−)-α-Bisabolol is a highly lipophilic compound that is almost insoluble in water, which limits its pharmaceutical applications [9,28]. In addition, its metabolic fate has not been extensively investigated in the previously reported metabolism studies of (−)-α-bisabolol. Thus, in order to obtain new and polar metabolites of **1**, biotransformation studies using microorganisms as biocatalysts were carried out. The fungus *Absidia coerulea* was selected to transform **1**, and the subsequent scale-up fermentation led to the isolation of seven new and four known hydroxylated metabolites (**2**–**12**, Figure 2). Moreover, hydroxylation of (−)-α-bisabolol using microorganisms could present a valuable method to generate oxygenated bisabolane-type sesquiterpenoids, which are potentially useful for pharmaceuticals and cosmetics.

## 2. Results and Discussion

A total of 15 microbial cultures were evaluated for their ability to metabolize (−)-α-bisabolol (**1**) using the usual two-stage fermentation procedure. Based on TLC analyses and control studies, it was observed that *Absidia coerulea*, *Aspergillus fumigatus*, *Cunninghamella elegans* var. *elegans* and *Mucor hiemalis* showed the ability to metabolize **1** (Supplementary Table S1). Among the four active strains, *A. coerulea* exhibited the highest transformational capability and produced a greater number of metabolites. Thus, *A. coerulea* was chosen for preparative-scale fermentation of **1** to produce sufficient quantities of metabolites for structural elucidation.

Seven new and four known metabolites were obtained (Figure 2). The four known metabolites were identified as (1*S*,7*S*)-4,7-dihydroxy-α-bisabolol (**6**) [29], (1*S*,3*R*,4*R*,7*S*)-3,4-dihydroxy-α-bisabolol (**9**) [17], 15-hydroxy-α-bisabolol (**11**) [30], and 11-hydroxy-T-muurolol (**12**) [31] by the combined analysis of NMR and IR spectroscopic data.

**Figure 3:**
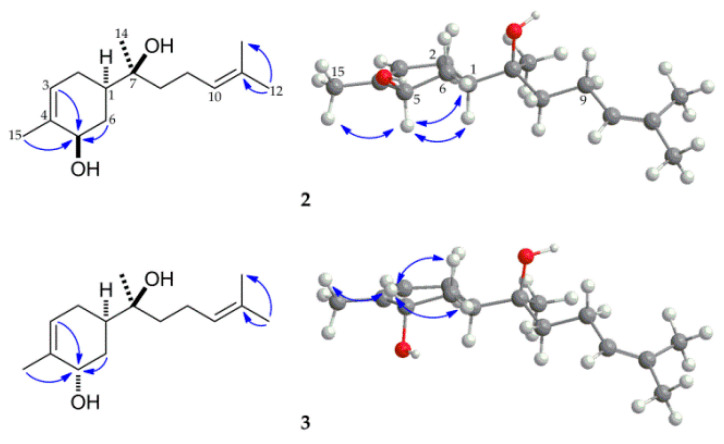
Selected HMBC (^1^H→^13^C) and NOE (^1^H↔^1^H) correlations of metabolites **2** and **3**.

Compound **2** was obtained as a colorless oil, and its molecular formula was deduced as C_15_H_26_O_2_ based on its HRESIMS analysis (*m/z* 261.1831, calcd for C_15_H_26_O_2_Na, 261.1830), which indicated that it was a monohydroxylated metabolite of **1**. The additional oxygen-bearing secondary carbon resonating at δ_C_ 71.0 was considered to be C-5 after comparing the ^13^C NMR data of related compounds with the hydroxyl group substituted at position C-5 [32]. Moreover, this was further supported by the long-range correlations of H-3 (δ_H_ 5.47), H-6 (δ_H_ 2.25, 1.35) and H-15 (δ_H_ 1.76), with C-5 (δ_C_ 71.0) in the HMBC spectrum of **2** (Figure 3). The relative configuration of 5-OH was deduced as β by the NOE (nuclear Overhauser effect) correlations among H-5 (δ_H_ 4.17) and H-1 (δ_H_ 1.72), H-6eq (δ_H_ 2.25), and H-15 (δ_H_ 1.76) (Figure 3). On the basis of the above analysis and comparison with previously reported NMR data of related compounds [32,33], compound **2** was identified as (1*R*,5*R*,7*S*)-5-hydroxy-α-bisabolol.

Compound **3** was obtained as a colorless oil. Its HRESIMS showed an [M + Na]^+^ peak at *m/z* 261.1830 (calcd for C_15_H_26_O_2_Na, 261.1830), which established a molecular formula of C_15_H_26_O_2_, indicating that it was a monohydroxylated derivative of **1**. The presence of the hydroxyl-bearing methine proton at δ_H_ 4.05 (1H, br s) with corresponding carbon at δ_C_ 68.6 was identified based on the ^1^H and ^1^^3^C NMR spectral analysis of **3** with the help of an HSQC experiment (Supplementary Figure S15). Compounds **2** and **3** had similar NMR spectral data, suggesting that they were epimers. The location of the hydroxyl group at the C-5 position was supported by the long-range correlations from H-3 (δ_H_ 5.56), H-6 (δ_H_ 1.44, 2.03), and H-15 (δ_H_ 1.79) to C-5 (δ_C_ 68.6) in the HMBC spectrum of **3**. The correlation between H-5 and H-1 was not observed in the NOE spectrum of **3** (Figure 3) indicating that the hydroxyl group at C-5 position was α-oriented, and the broad singlet peak of H-5 in its ^1^H NMR spectrum also supported this elucidation. Therefore, compound **3** was established as (1*R*,5*S*,7*S*)-5-hydroxy-α-bisabolol.

**Table 1:** ^1^H-NMR data of metabolites **2**–**5**, **7**, **8** and **10**.

Position	Compound						
	2 ^a^	3 ^a^	4 ^b^	5 ^b^	7 ^b^	8 ^b^	10 ^b^
1	1.72, 1H, m	1.79, 1H, m	1.77, 1H, m	-	1.57, 1H, m	1.72, 1H, m	1.53, 1H, tt (11.8, 2.8)
2	1.98, 1H, m	2.06, 1H, m	1.97, 2H, m	2.14, 1H, m	1.98, 1H, m	1.78, 1H, d (3.0)	1.89, 1H, d (11.8)
	1.86, 1H, m	1.77, 1H, m		1.92, 1H, m	1.79, 1H, m	1.73 1H, dt (3.0, 13.7)	1.18, 1H, m
3	5.47, 1H, m	5.56, 1H, dt (5.4, 1.5)	5.47, 1H, m	5.30, 1H, m	5.37, 1H, m	3.63, 1H, br s	3.53, 1H, dd (11.6, 4.0)
5	4.17, 1H, brs	4.05, 1H, brs	4.19, 1H, m	2.21, 2H, m	1.99, 2H, m	1.74, 1H, m 1.55, 1H, m	1.80, 1H, dt (3.1, 12.9) 1.42, 1H, td (12.9, 3.1)
6	2.25, 1H, ddt (12.0, 6.0, 2.3)	2.03, 1H, m	2.26, 1H, dd (11.8, 5.6)	1.65, 1H, dd (5.8, 12.8)	1.91, 1H, m	1.60, 1H, m	1.72, 2H, m
	1.35, 1H, td (12.0, 10.0)	1.44, 1H td (13.0, 3.8)	1.28, 1H, m	1.76, 1H, ddt (12.8, 5.8, 2.0)	1.29, 1H, m	1.42, 1H, m	
8	1.51, 2H, m	1.52, 2H, m	1.85, 1H, m 1.62, 1H, m	2.29, 1H, dt (12.2, 7.5), 1.57, 1H, dd (12.2, 7.5)	1.52, 2H, m	1.50, 2H, m	1.49, 2H, m
9	2.04, 2H, m	2.07, 2H, m	1.83, 2H, m 1.78, 2H, m	1.90, 2H, m 1.85, 2H, m	2.12, 2H, ddd (7.8)	2.05, 2H, m	2.04, 2H, m
10	5.12, 1H, tq (7.0, 1.4)	5.13, 1H, tt (7.3, 1.3)	3.68, 1H, dd (9.2, 5.7)	3.76, 1H, dd (10.6, 5.4)	5.42, 1H, tq (7.2, 1.6)	5.13, 1H, t (7.0)	5.12, 1H, t (6.6)
12	1.69, 3H, s	1.69, 3H, s	1.11, 3H, s	1.13, 3H, s	4.00, 3H, s	1.69, 3H, s	1.69, 3H, s
13	1.62, 3H, s	1.62, 3H, s	1.21, 3H, s	1.23, 3H, s	1.68, 3H, s	1.63, 3H, s	1.62, 3H, s
14	1.13, 3H, s	1.12, 3H, s	1.13, 3H, s	1.16, 3H, s	1.12, 3H, s	1.14, 3H, s	1.14, 3H, s
15	1.76, 3H, s	1.79, 3H, s	1.75, 3H, s	1.70, 3H, s	1.65, 3H, s	1.26, 3H, s	1.18, 3H, s

Coupling constants (*J*) are given in Hz; ^a^ Spectra recorded at 500 MHz in CDCl_3_; ^b^ Spectra recorded at 400 MHz in CDCl_3_.

**Figure 4:**
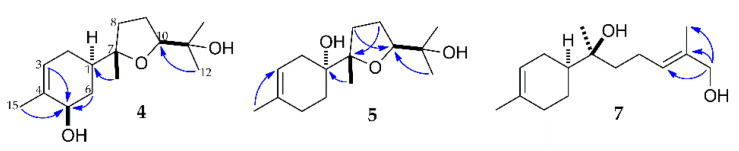
Selected HMBC (^1^H→^13^C) correlations of metabolites **4**, **5**, and **7**.

Compound **4** was obtained as colorless oil. Its molecular formula was determined to be C_15_H_26_O_3_ by the [M + Na]^+^ peak at *m/z* 277.1782 (calcd for C_15_H_26_O_3_Na, 277.1780) from its HRESIMS spectrum, indicating that it was a di-oxygenated metabolite of **1**. Similar resonance signals consisting of one methyl group, two methylene groups, three methine groups (including one oxygen-bearing methine), and one quaternary carbon of the cyclohexane system were observed for **4** in comparison to the ^1^H and ^13^C NMR data of **2** (Table 1 and Table 2), suggesting the presence of one hydroxyl group at the C-5 position. In addition, the NMR spectral features of **4** were quite similar to those of bisabolol oxide B [34], except for the existence of the C-5 hydroxyl group. The presence of the tetrahydrofuran ring was evident from the oxymethine signal at δ_H_ 3.68, two non-equivalent methylene signals at δ_H_ 1.85/1.62 and 1.83/1.78, together with three oxygenated carbon signals at δ_H_ 84.4, 86.1 and 70.5. These elucidations were supported by the long-range correlations from H-3/6/15 to C-5, from H-8/12/13 to C-11, from H-14 to C-1, and from H-9 to C-7 in the HMBC spectrum of **4** (Figure 4). The relative configurations of C-5 and C-10 were established to be *R* and *S*, respectively, according to the nuclear Overhauser correlations of H-5 with H-1, H-15 and H-6eq, along with no correlation between H-10 and H-14 in the NOESY spectrum of **4** (Supplementary Figure S23). After comparison with the previously reported data of bisabolol oxide B and xylcarpin D [34,35], compound **4** was established as (1*R*,5*R*,7*S*,10*S*)-5-hydroxy-bisabolol oxide B.

Compound **5** was obtained as a colorless oil. The molecular formula for **5** was deduced as C_15_H_26_O_3_ on the basis of the [M + Na]^+^ peak at *m/z* 277.1780 (calcd for C_15_H_26_O_3_Na, 277.1780) in its HRESIMS spectrum, indicating that it was also a di-oxygenated metabolite of **1**. Comparison of the ^1^H and ^13^C NMR data of **5** with those of **4** and bisabolol oxide B suggested that the double bond in the C-3(4) position remained and the double bond in the C-10(11) position was oxidized to form the tetrahydrofuran ring (Table 1 and Table 2) [17]. Similar to compound **4**, the configuration of C-10 was established as *S*, and no correlation between H-10 and H-14 was observed in the NOESY spectrum of **5**. With three methylene groups, one trisubstituted C=C group existed in the cyclohexane moiety of **5**, indicating that the oxygenated quaternary carbon should be located at the C-1 position. Moreover, the location was confirmed by the long-range correlation between H-14 (δ_H_ 1.16) and C-1 (δ_C_ 73.6) in its HMBC spectrum (Figure 4). In addition, the hydroxyl group at C-1 was supposed to be α-oriented according to its specific rotation, as 1α-OH exhibited a significantly negative rotation in the studies of β-bisabolol [36,37]. Thus, compound **5** was identified as (1*R*,7*S*,10*S*)-1-hydroxybisabolol oxide B.

Compound **7** was obtained as a colorless oil. The molecular formula for **7** was determined to be C_15_H_26_O_2_ according to the [M + Na]^+^ peak at *m/z* 261.1831 (calcd for C_15_H_26_O_2_Na, 261.1830) in its HRESIMS spectrum, indicating that it was a monohydroxylated metabolite of **1**. A notable change was observed in the ^1^H NMR spectrum of **7** compared to that of **1**, as one allylic methyl group signal was replaced with the hydroxymethylene group at δ_H_ 4.00. The significant downfield chemical shift of H-10 at δ_H_ 5.42 indicated that the hydroxyl group was attached to the C-12. This interpretation was supported by the long-range correlations from H-10/13 to C-12, and H-12 to C-10/11/13 in the HMBC spectrum of **7** (Figure 4). In addition, the geometry of the *Δ*^10^ double bond was assigned as *trans* configuration based on the correlations between H-12 (δ_H_ 4.00) and H-10 (δ_H_ 5.42), and H-13 (δ_H_ 1.68) in the NOE spectrum of **7**. Based on the above analysis and comparison with previously reported NMR data of 7,13-dihydroxybisabol-2,10-diene [38], compound **7** was identified as 12-hydroxy-α-bisabolol.

**Figure 5:**
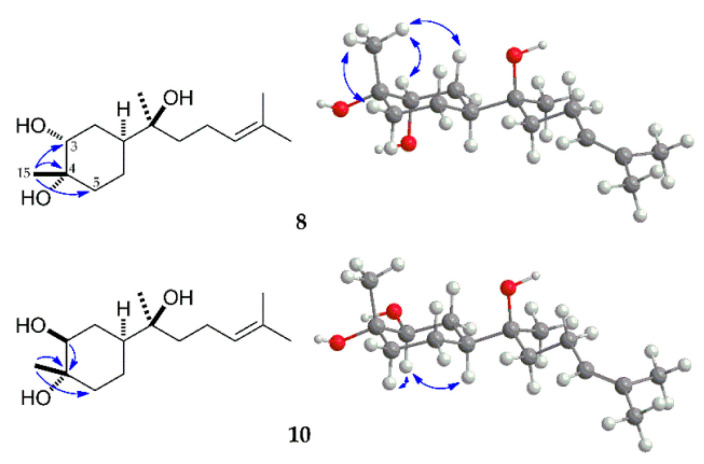
Selected HMBC (^1^H→^13^C) and NOE (^1^H↔^1^H) correlations of metabolites **8** and **10**.

Compound **8** was obtained as a colorless oil. The molecular formula for **8**, C_15_H_28_O_3_, was deduced from the [M + Na]^+^ peak at *m/z* 279.1937 (calcd for C_15_H_28_O_3_Na, 279.1936) in its HRESIMS, indicating that **8** was a dihydroxylated derivative of the parent compound **1**. The ^1^H and ^13^C NMR spectra of **8** showed signals of four *tert*-methyl groups, five methylene groups, three methine groups, and three quaternary carbons. The olefinic proton and carbon signals at C-3 disappeared with the appearance of one oxygen-bearing proton signal at δ_H_ 3.63 and two oxidized carbon signals at δ_C_ 74.0 and 70.9, which indicated that the double bond at the C-3(4) position was oxidized to a vicinal-diol, suggesting the structure of **8** was 3,4-dihydroxy-α-bisabolol. It was confirmed by the correlations from H-15 to C-3 and C-5 in the HMBC spectrum of **8** (Figure 5). The 3-OH group was deduced to be α-oriented based on the appearance of a broad singlet proton signal for H-3 in its ^1^H-NMR spectrum. The stereochemistry of **8** was indicated as 3α,4α-diol based on the nuclear Overhauser correlations among H-15 with H-3eq, H-5eq, H-2ax in the NOE spectrum (Figure 5). Based on the above analysis, compound **8** was characterized as (1*S*,3*R*,4*S*,7*S*)-3,4-dihydroxy-α-bisabolol.

Compound **10** was obtained as a colorless oil. Its molecular formula was deduced as C_15_H_28_O_3_ on the basis of the [M + Na]^+^ peak at *m/z* 279.1935 (calcd for C_15_H_28_O_3_Na, 279.1936) in its HRESIMS. The ^1^H and ^13^C NMR spectral data of **10** were very similar to those of **8**, indicating that **10** is a stereoisomer of **8**. The orientation of H-3 was determined to be axial from the large coupling constant (*J* = 11.6 Hz) of the H-3 signal in its ^1^H NMR [17,39]. The chemical shift of C-15 in compound **10** was shown in a higher magnetic field than that of **8** (Table 2), which was dependent on the γ-gauche effect of the equatorial γ-OH [17], suggesting that the methyl group at the C-4 position existed in the axial orientation. The configuration of **10** was further confirmed by the correlations among H-3 to H-5ax, H-1ax and H-2eq, without the correlation between H-3 and H-15 in the NOE spectrum (Figure 5). Therefore, based on the above analysis and comparison with the NMR data of (1*S*,3*S*,4*S*,7*S*,10*S*)-3,4-dihydroxy-bisabolol oxide B [17], the structure of **10** was assigned (1*S*,3*S*,4*S*,7*S*)-3,4-dihydroxy-α-bisabolol.

All the metabolites obtained during this study were more polar than their parent compound (−)-α-bisabolol (**1**) and proposed to have higher aqueous solubility. In order to confirm the improvement in solubility, a modified kinetic solubility test was carried out using the stock solutions of compounds **1**–**12** (20 mM in dimethyl sulfoxide). White turbidity was observed for **1** after diluting 10 μL of its stock solution with 100 μL water, while no precipitates were found for the metabolites **2**–**12**, and thus their kinetic solubility was expected to be higher than 1.82 mM [40]. This was confirmed by measuring the light transmittance of the diluted solutions. For example, compounds **2**, **5**, **8** and **11**, showed 90% transmittance at the wavelength of 390 to 650 nm, whereas, (−)-α-bisabolol showed less than 50% transmittance in this wavelength range (Supplementary Figure S2) [41]. In addition, the solubility of **1** was determined to be 0.26 mM based on the peak area shown in its HPLC chromatogram, as the area under a peak is a measure of the concentration of the compound it represents [42].

**Figure 6:**
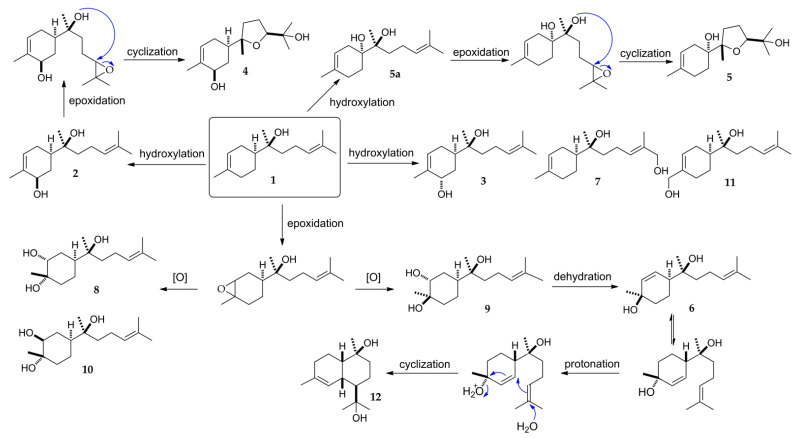
A proposed metabolic pathway of (−)-α-bisabolol (**1**) transformed by *A. coerulea*.

In the biotransformation studies of (−)-α-bisabolol carried out by Miyazawa and colleagues using the fungus *G. cingulata*, all the metabolites were identified as oxidized products in the carbon-carbon double bond positions [17]. Their major metabolites (1S,3R,4R,7S,10S)- and (1S,3S,4S,7S,10S)-3,4-dihydroxy-bisabolol oxide B were proposed to have been further transformed from the currently isolated metabolites (1*S*,3*R*,4*R*,7*S*)- and (1*S*,3*S*,4*S*,7*S*)-3,4-dihydroxy-α-bisabolol (**9** and **10**). In the present study, four hydroxylated metabolites were also obtained (**2**, **3**, **7**, **11**) without modification of any double bonds in the skeleton of α-bisabolol. The metabolic pathway in the biotransformation of **1** by *A. coerulea* was proposed (Figure 6) based on the relevant reaction procedures described in the previously reported literatures [17,43,44,45]. During the transformation process, α-bisabolol was directly hydroxylated to form compounds **2**, **3**, **5a**, **7**, and **11**. Next, epoxidation of the double bond in the prenyl side chain of compounds **2** and **5a** followed by a cyclization process led to the formation of compounds **4** and **5**. Metabolites **8**–**10** were formed through oxidation of the double bond existing in the cyclohexene system. In addition, it was thought that metabolite **6** was further converted from **9** through dehydration reaction. The hydroxyl group of **6** at C-4 position was postulated to undergo protonation and dehydration leading to a ring closure, followed by a hydration process to form the cadinane-type bicyclic sesquiterpene **12**.

## 3. Materials and Methods

### 3.1. General Experimental Procedures

Optical rotations were measured with a 343 Plus polarimeter (Perkin Elmer, Waltham, MA, USA). UV spectra were recorded on a JASCO V-530 spectrophotometer, and IR spectra were obtained on a JASCO FT/IR-300E spectrometer (Jasco, Tokyo, Japan). NMR experiments were recorded using a Varian Unity Inova 500 spectrometer (Varian, Palo Alto, CA, USA) and a Bruker Avance III HD 400 spectrometer (Bruker, Billerica, MA, USA) with TMS as an internal standard. HRESIMS analysis was performed on a Waters Synapt G2 mass spectrometer (Waters, Milford, MA, USA). TLC was carried out on precoated silica gel 60 F_254_ glass plates (Merck, Darmstadt, Germany). Visualization of the silica gel TLC was performed using an anisaldehyde-H_2_SO_4_ spray reagent. The adsorbent used for column chromatography was silica gel 70–230 mesh. HPLC was performed on a Waters 600E Multisolvent Delivery System (Waters, Milford, MA, USA) connected to a Waters 996 Photodiode Array Detector using Zorbax SB-CN (10 μm, 9.4 × 250 mm) and Chiralpak AD-H (5 μm, 250 × 4.6 mm) columns.

### 3.2. Materials and Microorganisms

(−)-α-Bisabolol (**1**) was purchased from Sigma-Aldrich (St. Louis, MO, USA). All the ingredients for microbial media including D-glucose, peptone, yeast extract, malt extract, and potato dextrose medium were purchased from Becton, Dickinson and Company (Sparks, MD, USA).

All the microorganisms were obtained from the Korean Collection for Type Cultures (KCTC). The microorganisms used for preliminary screening were as follows: *Absidia coerulea* KCTC 6936, *Alternaria alternata* 6005, *Aspergillus fumigatus* 6145, *Cunninghamella elegans* var. *elegans* 6992, *Filobasidium neoformans* 7902, *Fusarium merismoides* 6153, *Gliocladium deliquescens* 6173, *Glomerella cingulata* 6075, *Hormoconis resinae* 6966, *Kluyveromyces marxianus* 7155, *Microbacterium lacticum* 9230, *Mortierella ramanniana* var. *angulispora* 6137, *Mucor hiemalis* 26779, *Penicillium chrysogenum* 6933, *Trichoderma koningii* 6042.

Fermentation experiments were performed in three types of media: *A. coerulea*, *A. alternata*, *A. fumigatus*, *M. hiemalis*, *P. chrysogenum* and *T. koningii* were incubated on malt medium (malt extract 20 g/L, D-glucose 20 g/L, peptone 1 g/L); *F. neoformans*, *K. marxianus*, and *M. lacticum* were cultured on yeast-malt medium (D-glucose 10 g/L, peptone 5 g/L, malt extract 3 g/L, and yeast extract 3 g/L); other microorganisms were cultured on potato dextrose medium (potato dextrose broth 24 g/L).

### 3.3. Screening Procedures

Microbial metabolism studies were carried out according to the two-stage procedure [46,47]. In the screening studies, the actively growing microbial cultures were inoculated in 250 mL flasks containing 50 mL of a suitable medium and incubated with gentle agitation (200 rpm) at 25 °C in a temperature-controlled shaking incubator. After inoculation for 24 h, 100 μL of ethanol solution (20 mg/mL) of **1** was added to each flask, and the transformation was continued under the same condition for an additional seven days. Both substrate and culture controls were carried out under the same conditions. Sampling and TLC monitoring were performed at an interval of 24 h. Culture controls consisted of fermentation cultures in which the microorganisms were grown without the addition of **1**.

### 3.4. Extraction and Isolation of Metabolites

Scale-up fermentations were carried out under the same condition with twenty-six 1L flasks, each containing 200 mL media and 520 mg of **1** dissolved in EtOH (20 mg/mL) was evenly distributed among flasks. The cultures were extracted with equal volume of EtOAc (5.2 L × 3) after seven day incubation and the organic layers were combined and concentrated under reduced pressure [46]. The crude EtOAc extracts (895 mg) were separated by silica gel column chromatography using *n*-hexane-EtOAc gradient solvent system (10:1→2:3) to give 10 fractions. Fraction 3 was subjected to HPLC with 50% MeOH as elution solvent to give compound **12** (6.02 mg, 1.08%) and subfraction 3-1. Subfraction 3-1 was purified by HPLC using 45% MeOH isocratic solvent system to afford compound **5** (2.74 mg, 0.46%). Fraction 5 was chromatographed by HPLC using 45% MeOH isocratic solvent system to give compounds **4** (6.35 mg, 1.07%), **6** (3.42 mg, 0.61%), **11** (15 mg, 2.69%), **2** (17 mg, 3.05%) and subfraction 5-5. Subfraction 5-5 was further chromatographed by HPLC with a chiral column using 100% MeOH as mobile phase to afford compounds **7** (4.2 mg, 0.75%) and **3** (2.45 mg, 0.44%). Fraction 10 was chromatographed by HPLC using 35% MeOH isocratic solvent system to afford compound **8** (2.85 mg, 0.48%) and subfraction 10-4. Subfraction 10-4 was further subjected to HPLC with a chiral column using 100% MeOH as mobile phase to give compounds **10** (6.09 mg, 1.03%) and **9** (2.45 mg, 0.41%).

### 3.5. Spectroscopic Data of Metabolites

#### (1*R*,5*R*,7*S*)-5-hydroxy-α-bisabolol (**2**)

Colorless oil. [α${]}_{D}^{20}$: −11.4° (*c* 0.048, MeOH). UV λ_max_ (MeOH): 200 nm. IR *ν*_max_ cm^−1^: 3353, 2966, 2920, 1656, 1443, 1032, 965. ^1^H-NMR (CDCl_3_, 500 MHz): see Table 1; ^13^C NMR (CDCl_3_, 125 MHz): see Table 2; HRESIMS: *m/z* 261.1831 [M + Na]^+^ (calcd. for C_15_H_26_O_2_Na, 261.1830).

#### (1*R*,5*S*,7*S*)-5-hydroxy-α-bisabolol (**3**)

Colorless oil. [α${]}_{D}^{20}$: −89.1° (*c* 0.03, MeOH). UV λ_max_ (MeOH): 204 nm. IR *ν*_max_ cm^−1^: 3373, 2969, 2922, 2860, 1660, 1453, 1376, 1108, 1035, 929. ^1^H-NMR (CDCl_3_, 500 MHz): see Table 1; ^13^C NMR (CDCl_3_, 125 MHz): see Table 2; HRESIMS: *m/z* 261.1830 [M + Na]^+^ (calcd. for C_15_H_26_O_2_Na, 261.1830).

#### (1*R*,5*R*,7*S*,10*S*)-5-hydroxybisabolol oxide B (**4**)

Colorless oil. [α${]}_{D}^{20}$: −20° (*c* 0.08, MeOH). UV λ_max_ (MeOH): 198 nm. IR *ν*_max_ cm^−1^: 3396, 2971, 2920, 2871, 1659, 1546, 1376, 1056, 889. ^1^H-NMR (CDCl_3_, 400 MHz): see Table 1; ^13^C NMR (CDCl_3_, 100 MHz): see Table 2; HRESIMS: *m/z* 277.1782 [M + Na]^+^ (calcd. for C_15_H_26_O_3_Na, 277.1780).

#### (1*R*,7*S*,10*S*)-1-hydroxybisabolol oxide B (**5**)

Colorless oil. [α${]}_{D}^{20}$: −153.2° (*c* 0.006, MeOH). UV λ_max_ (MeOH): 200 nm. IR *ν*_max_ cm^−1^: 3405, 2971, 2931, 1712, 1455, 1378, 1059, 890. ^1^H-NMR (CDCl_3_, 400 MHz): see Table 1; ^13^C NMR (CDCl_3_, 100 MHz): see Table 2; HRESIMS: *m/z* 277.1780 [M + Na]^+^ (calcd. for C_15_H_26_O_3_Na, 277.1780).

#### 12-hydroxy-α-bisabolol (**7**)

Colorless oil. [α${]}_{D}^{20}$: −44.3° (*c* 0.037, MeOH). UV λ_max_ (MeOH): 206 nm. IR *ν*_max_ cm^−1^: 3363, 2921, 1677, 1451, 1013. ^1^H-NMR (CDCl_3_, 400 MHz): see Table 1; ^13^C NMR (CDCl_3_, 100 MHz): see Table 2; HRESIMS: *m/z* 261.1831 [M + Na]^+^ (calcd. for C_15_H_26_O_2_Na, 261.1830).

#### (1*S*,3*R*,4*S*,7*S*)-3,4-dihydroxy-α-bisabolol (**8**)

Colorless oil. [α${]}_{D}^{20}$: −31.5° (*c* 0.086, MeOH). UV λ_max_ (MeOH): 196 nm. IR *ν*_max_ cm^−1^: 3393, 2931, 2871, 1710, 1457, 1373, 1037, 909, 757. ^1^H-NMR (CDCl_3_, 400 MHz): see Table 1; ^13^C NMR (CDCl_3_, 100 MHz): see Table 2; HRESIMS: *m/z* 279.1937 [M + Na]^+^ (calcd. for C_15_H_28_O_3_Na, 279.1936).

#### (1*S*,3*S*,4*S*,7*S*)-3,4-dihydroxy-α-bisabolol (**10**)

Colorless oil. [α${]}_{D}^{20}$: −9.3° (*c* 0.048, MeOH). UV λ_max_ (MeOH): 198 nm. IR *ν*_max_ cm^−1^: 3398, 2939, 2870, 1709, 1378, 1069, 756. ^1^H-NMR (CDCl_3_, 400 MHz): see Table 1; ^13^C NMR (CDCl_3_, 100 MHz): see Table 2; HRESIMS: *m/z* 279.1935 [M + Na]^+^ (calcd. for C_15_H_28_O_3_Na, 279.1936).

### 3.6. Determination of Solubility

The solubility of compounds **1**–**12** was evaluated by the modified kinetic solubility assay [40,48]. Compounds **1**–**12** were dissolved in dimethyl sulfoxide (DMSO) to yield 20 mM stock solutions, then 10 μL of the compound stock solution was diluted with 100 μL water. After all the solutions were prepared, the vials were placed under room temperature and allowed to mix for 1 h. After this, the light transmittance was measured at a scan width of 5 nm over the range of 390 to 650 nm using a SpectraMax 190 Microplate reader (Molecular Devices, Sunnyvale, CA, USA). After centrifugation, the supernatant was analyzed by HPLC under 200 nm using a Zorbax SB-CN (250 × 10 mm) column with 60% methanol as mobile phase at a flow rate of 2 mL/min.

## 4. Conclusions

In this study, microorganisms were used as biocatalysts for the structural modification of (−)-α-bisabolol (**1**). After screening of 15 different microbial strains for their ability to metabolize xenobiotics, the filamentous fungus *A. coerulea* KCTC 6936 was selected for transformation of (−)-α-bisabolol according to its higher catalytic capability. Eleven hydroxylated metabolites including seven previously unreported compounds (**2**–**5**, **7**, **8**, **10**) were obtained from the culture of *A. coerulea* incubated with (−)-α-bisabolol. Among them, compounds **9** and **10** were proposed to be the transformation intermediates for the production of (1S,3R,4R,7S,10S)- and (1S,3S,4S,7S,10S)-3,4-dihydroxy-bisabolol oxide B. This evidence indicated that biocatalytic oxidation by *A. coerulea* is a feasible and effective approach to obtain more polar compounds which can be used as reference standards for the detection and identification of the metabolic products of (−)-α-bisabolol in a mammalian system. In addition, all of the isolated metabolites showed increased aqueous solubility compared with the parent compound **1**, and further studies are needed to evaluate the biological activities of the identified metabolites and find microorganisms which can produce the bioactive metabolites in higher yields.

## Figures and Tables

**Table 2:** ^13^C-NMR data of metabolites **2**–**5**, **7**, **8** and **10**.

Position	Compound						
	2 ^a^	3 ^a^	4 ^b^	5 ^b^	7 ^b^	8 ^b^	10 ^b^
1	42.1	36.8	43.5	73.6	43.0	39.3	45.6
2	27.1	27.1	27.3	32.3	27.0	29.9	32.1
3	123.7	125.3	123.8	117.9	120.5	74.0	77.4
4	136.6	134.4	136.6	134.0	134.2	70.9	74.0
5	71.0	68.6	71.1	26.7	31.0	33.6	38.4
6	33.8	32.0	34.9	28.5	23.3	21.3	23.6
7	73.9	73.8	84.4	88.0	74.3	74.3	73.9
8	39.9	40.2	35.5	32.2	39.8	39.5	39.8
9	22.1	22.1	26.4	26.3	21.7	22.2	22.2
10	124.3	124.4	86.1	87.9	126.2	124.5	124.2
11	131.9	131.8	70.5	70.4	134.9	131.9	132.1
12	25.7	25.7	24.0	24.0	68.9	25.7	25.8
13	17.7	17.7	27.7	27.8	13.7	17.7	17.7
14	23.4	23.3	23.5	22.9	23.2	24.2	24.1
15	18.8	20.8	18.8	23.5	23.4	27.5	18.9

^a^ Spectra recorded at 125 MHz in CDCl_3_; ^b^ Spectra recorded at 100 MHz in CDCl_3_.

## Data Availability

Data is contained within the article and Supplementary Materials.

## Supplementary Materials

The following supporting information can be downloaded online. Table S1: Screening for the microorganisms that transform (−)-α-bisabolol (**1**); Figure S1: Chemical structures of nootkatone, 13-hydroxynootkatone, caryophyllene oxide and 14-hydroxymethyl caryophyllene oxide; Figure S2: Light transmittance of the diluted solutions of (−)-α-bisabolol (**1**) and its metabolites in water; Figure S3: Calibration of measurement for solubility of (−)-α-bisabolol (**1**); Figures S4–S62: NMR and HRESIMS spectra of compounds **1**–**12**.

## Sample Availability

Samples of the compounds are not available from the authors.
