# Supplementary material for: Biotransformation of (−)-α-Bisabolol by Absidia coerulea
Source: Molecules. 2022 Jan 27;27(3):881. doi: 10.3390/molecules27030881 (PMC8840173; doi:10.3390/molecules27030881)
Supplement: Supplementary file 1 [file molecules-27-00881-s001.zip › molecules-1559459-supplementary.pdf]

molecules

Article

Biotransformation of (−)-*α*-Bisabolol by *Absidia coerulea*

Jisu Park [†,‡], Fubo Han [‡] and Ik-Soo Lee *

College of Pharmacy, Chonnam National University, Gwangju 61186, Korea; qkrwltn9410@naver.com (J.P.); hanfubo0306@gmail.com (F.H.)
* Correspondence: islee@chonnam.ac.kr; Tel.: +82-62-530-2932
† Current address: Advanced Radiation Technology Institute, Korea Atomic Energy Research Institute, Jeongeup-si 56212, Korea
‡ These authors contributed equally to this work.

Table S1. Screening for the microorganisms that transform (-)-*α*-bisabolol (**1**).

Microorganism	Capability*	Microorganism	Capability*
A. alternata	-	*H. resinae*	-
A. coerulea	+++	*K. marxianus*	-
A. fumigatus	+	*M. hiemalis*	++
C. elegans var. *elegans*	++	*M. lacticum*	-
F. neoformans	-	*M. ramanniana* var. *angulispora*	-
F. merismoides	-	*P. chrysogenum*	-
G. cingulata	-	*T. koningii*	-
G. deliquescens	-		

* Capability of transformation of (-)-α-bisabolol (**1**): (-) no transformation; (+) lower yield of metabolites; (++) higher yield of metabolites; (+++) highest yield of metabolites.

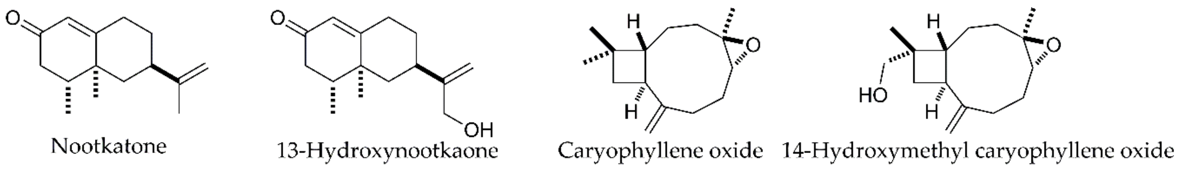

Figure S1. Chemical structures of nootkatone, 13-hydroxynootkatone, caryophyllene oxide and 14-hydroxymethyl caryophyllene oxide.

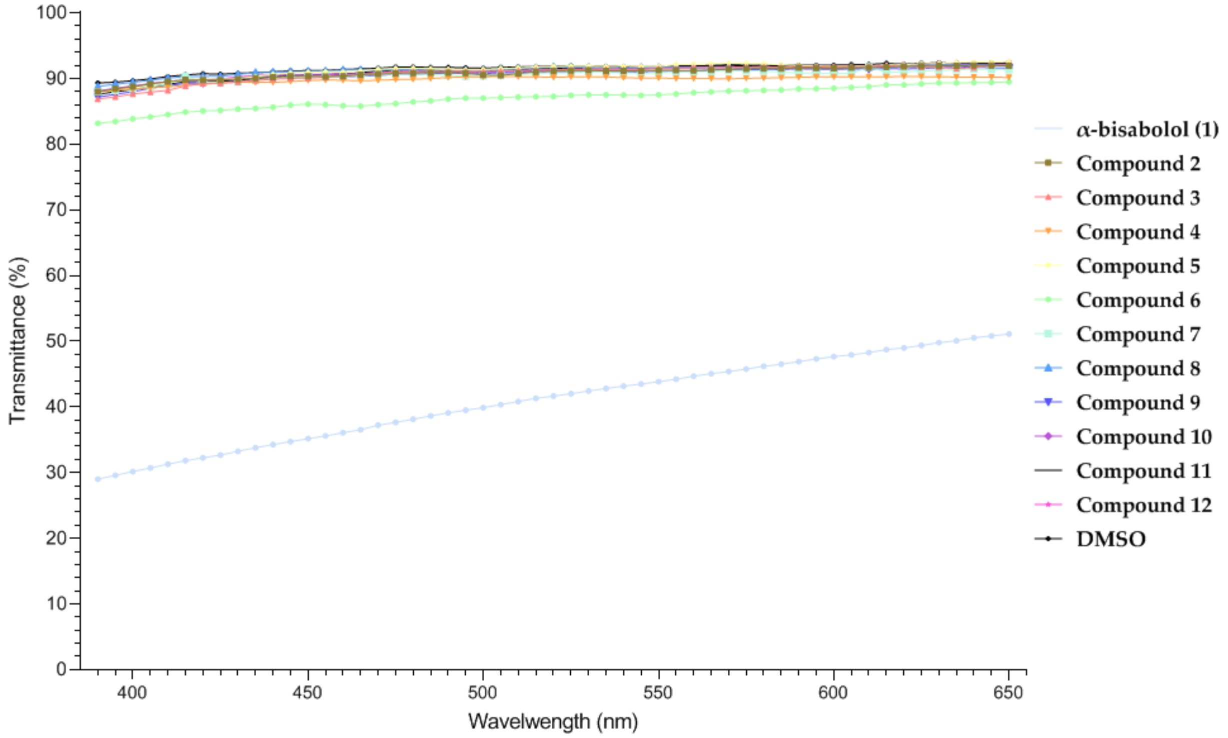

Figure S2. Light transmittance of the diluted solutions of (-)-α-bisabolol (1) and its metabolites in water.

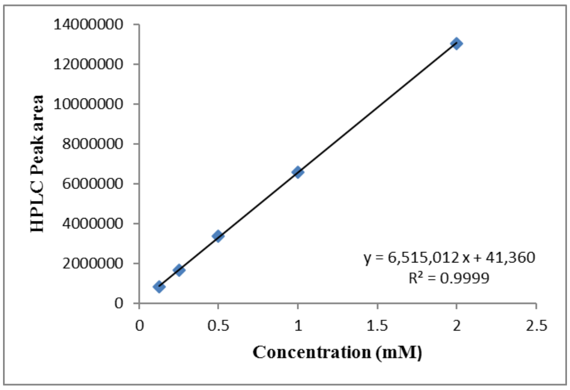

Figure S3. Calibration of measurement for solubility of (-)-α-bisabolol (**1**).

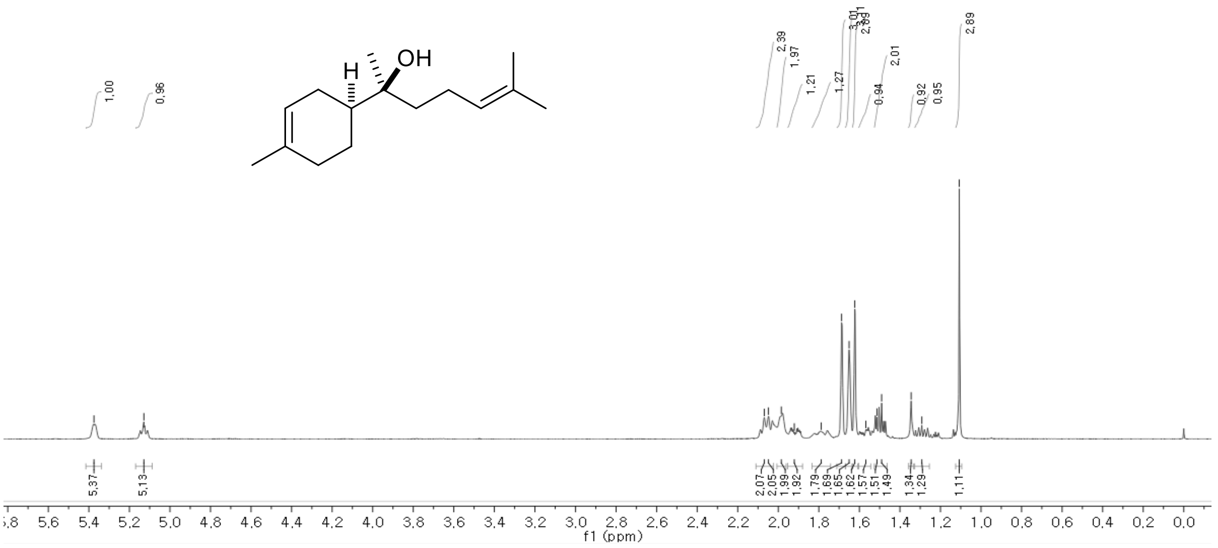

Figure S4. ^{1}H NMR spectrum of compound **1** recorded at 400 MHz in $CDCl_3$

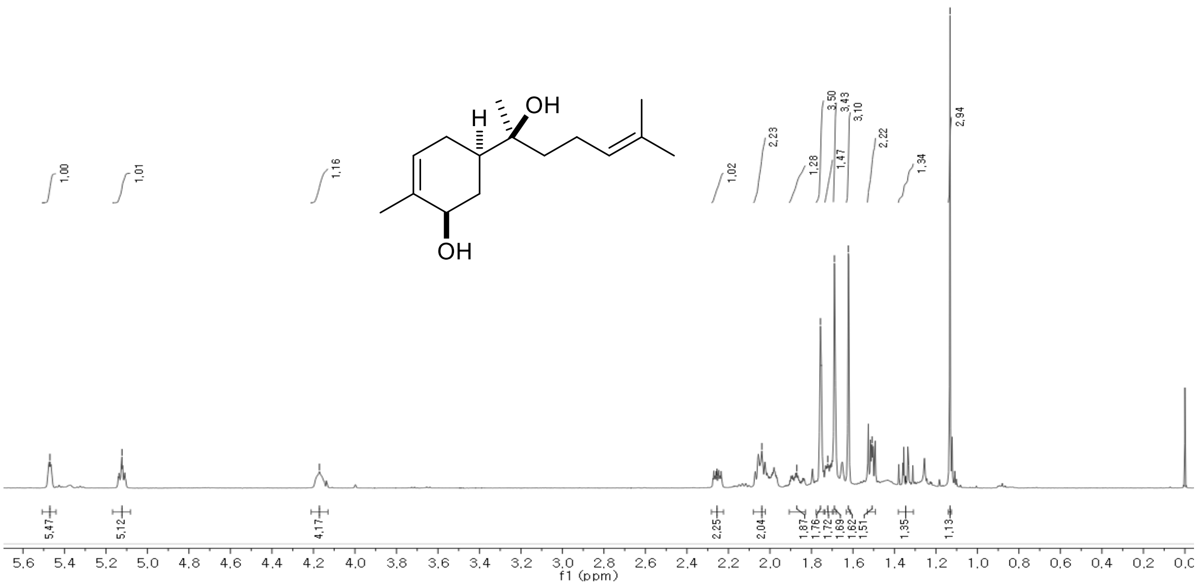

Figure S5. ^{1}H NMR spectrum of compound **2** recorded at 500 MHz in $CDCl_3$.

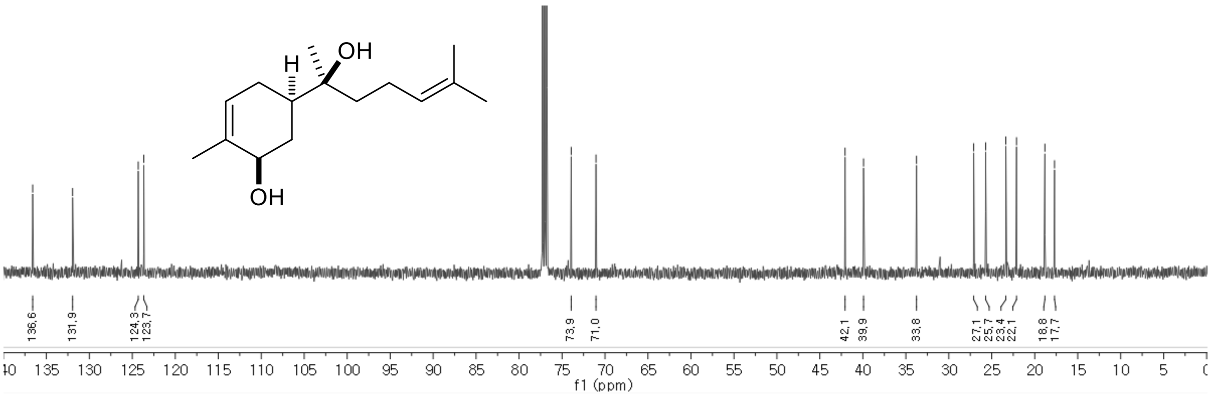

Figure S6. ^{13}C NMR spectrum of compound **2** recorded at 125 MHz in $CDCl_3$.

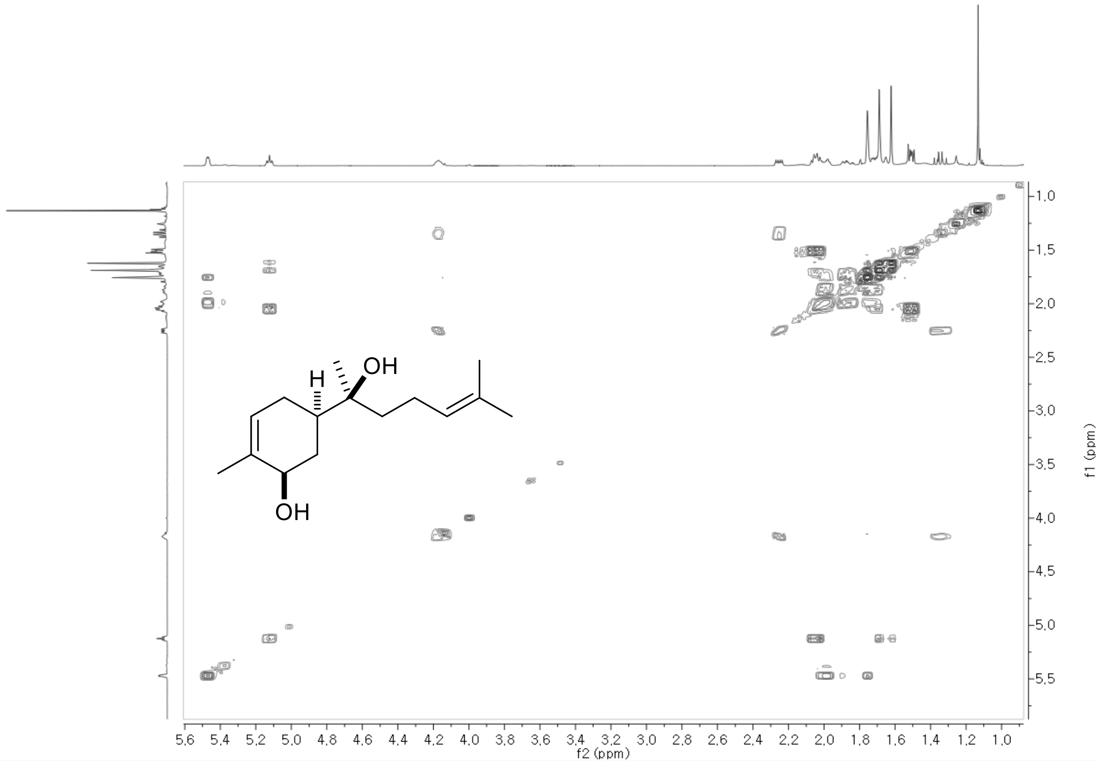

Figure S7. COSY spectrum of compound **2** recorded at 125 MHz in $CDCl_3$.

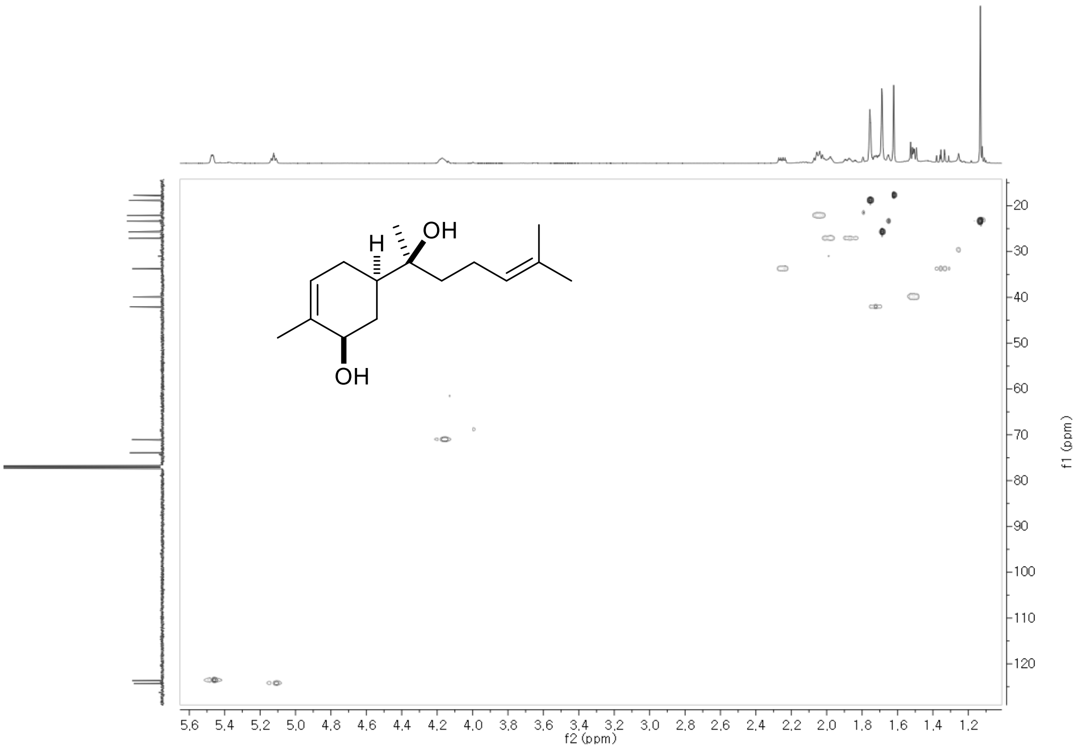

Figure S8. HSQC spectrum of compound **2** recorded at 500 MHz in $CDCl_3$.

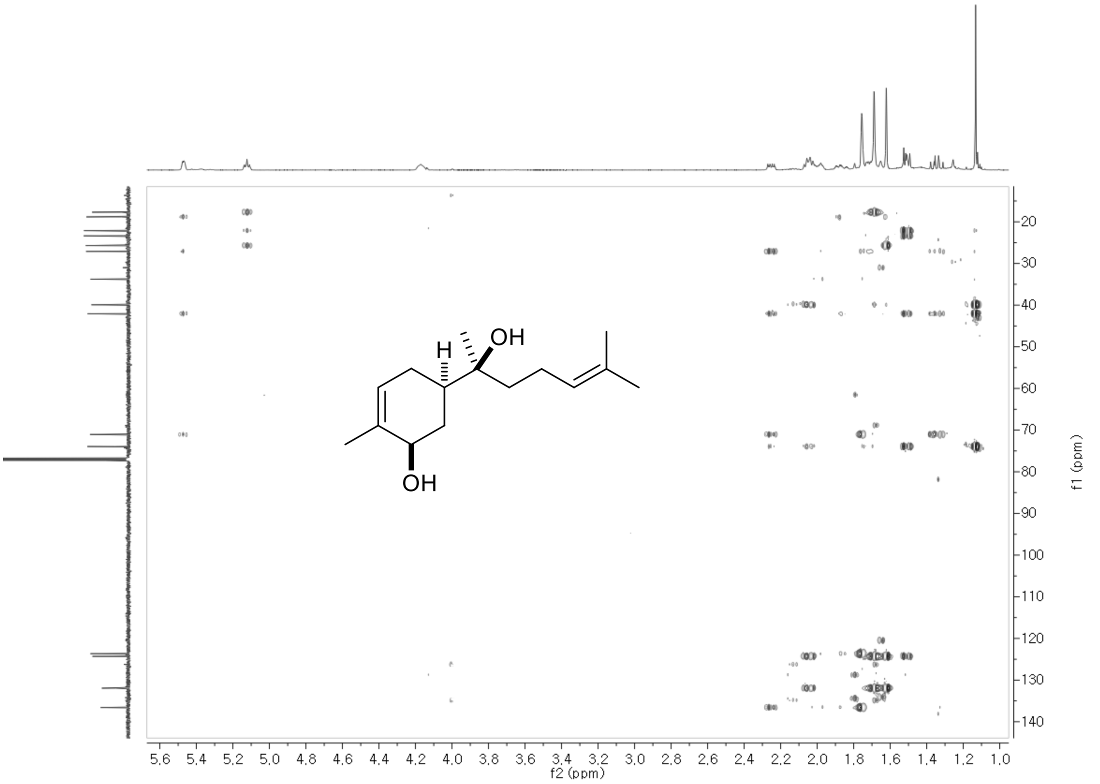

Figure S9. HMBC spectrum of compound **2** recorded at 500 MHz in CDCl3.

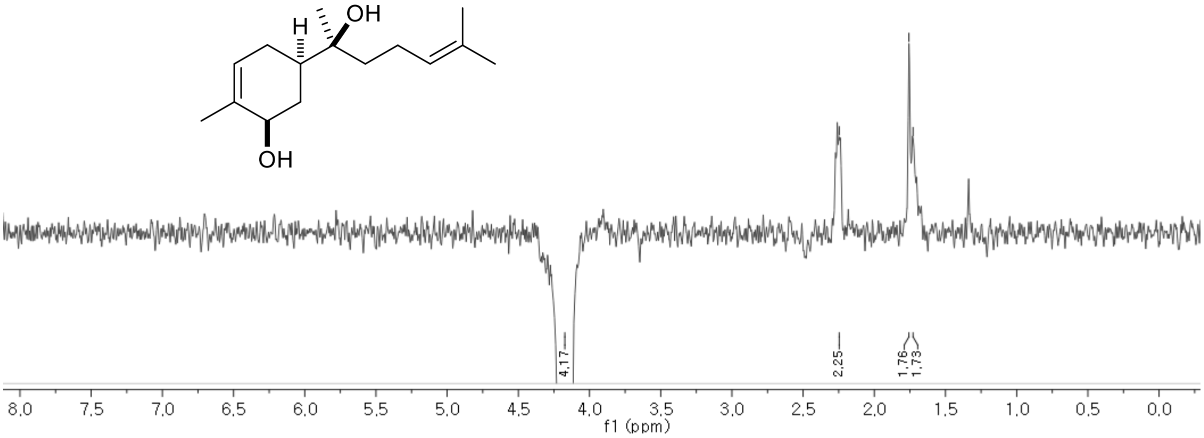

Figure S10. NOE spectrum of compound **2** recorded at 500 MHz in CDCl3.

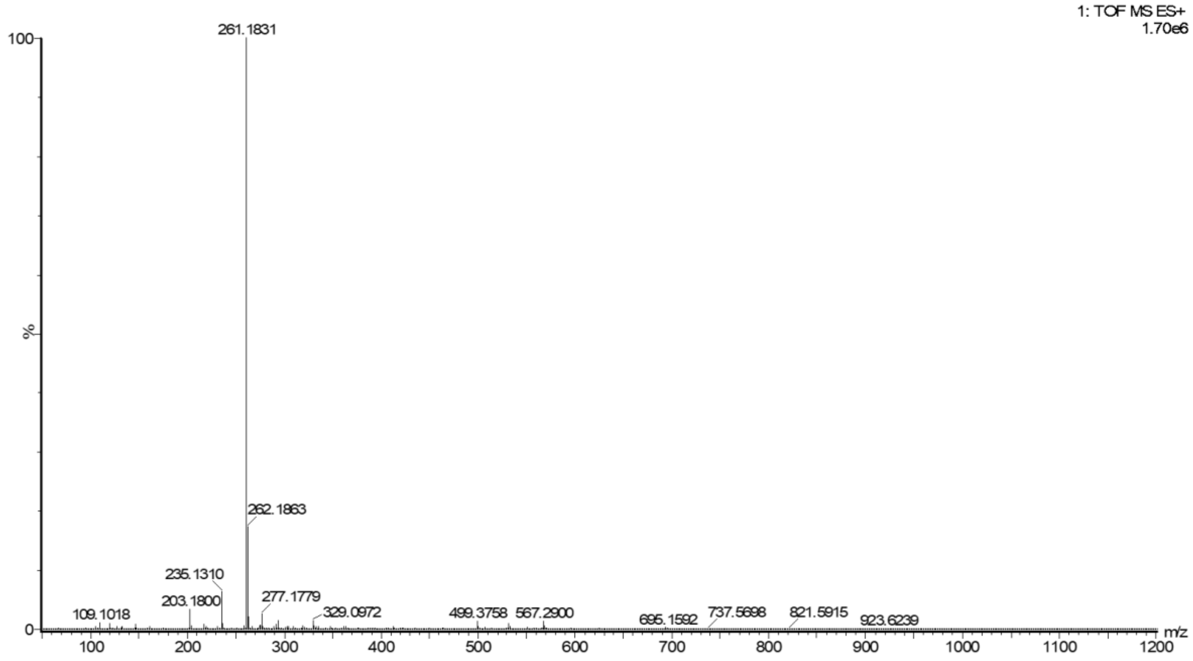

Figure S11. The HRESIMS spectroscopic data of compound **2.**

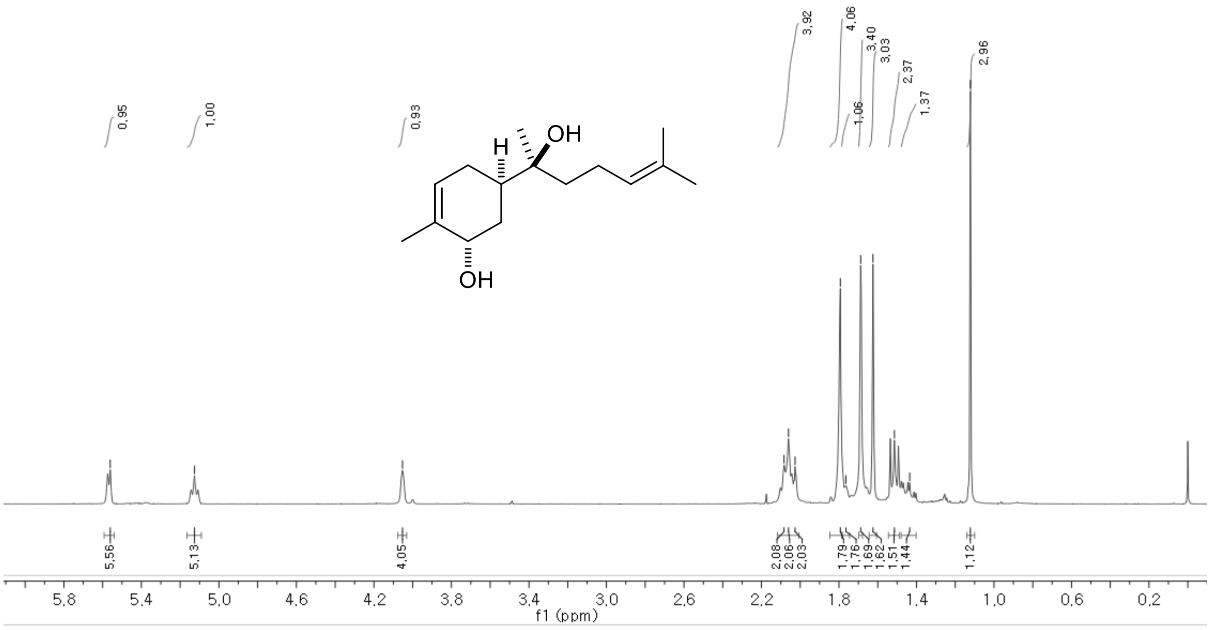

Figure S12. ^{1}H NMR spectrum of compound **3** recorded at 500 MHz in $CDCl_3$.

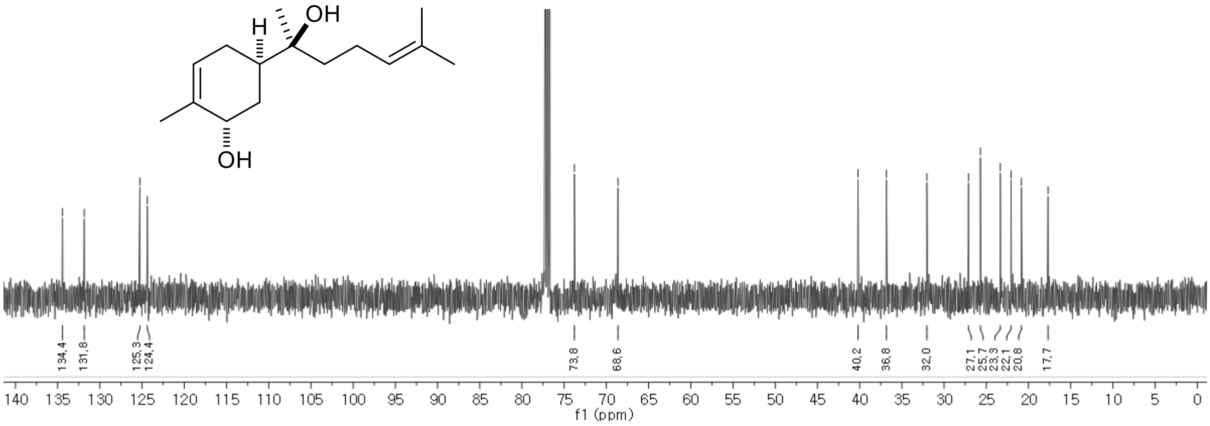

Figure S13. ^{13}C NMR spectrum of compound **3** recorded at 125 MHz in $CDCl_3$.

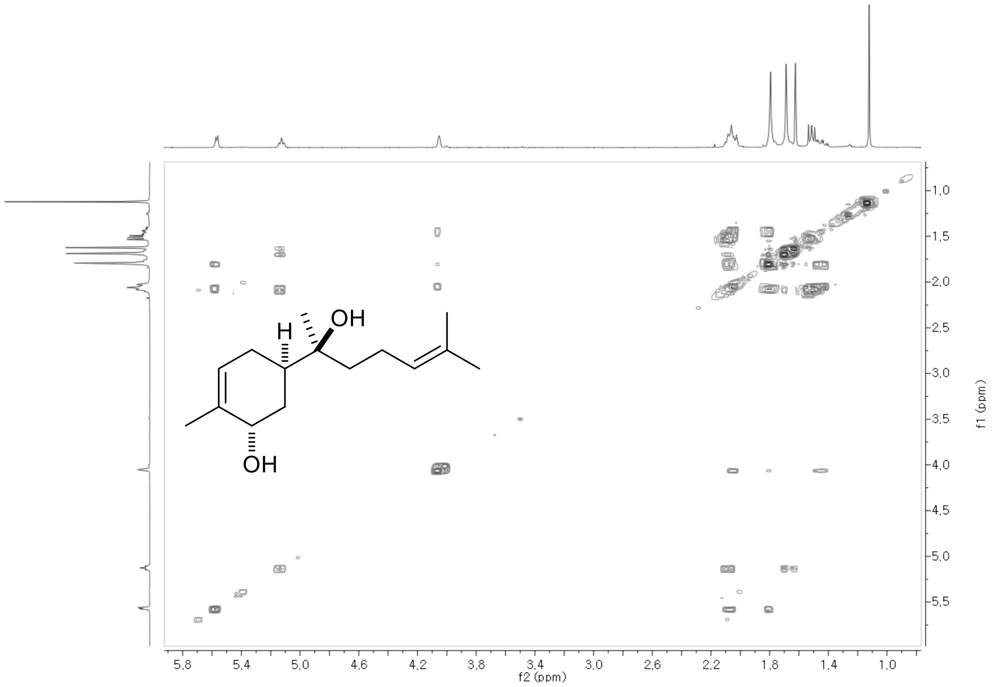

Figure S14. COSY spectrum of compound **3** recorded at 500 MHz in $CDCl_3$.

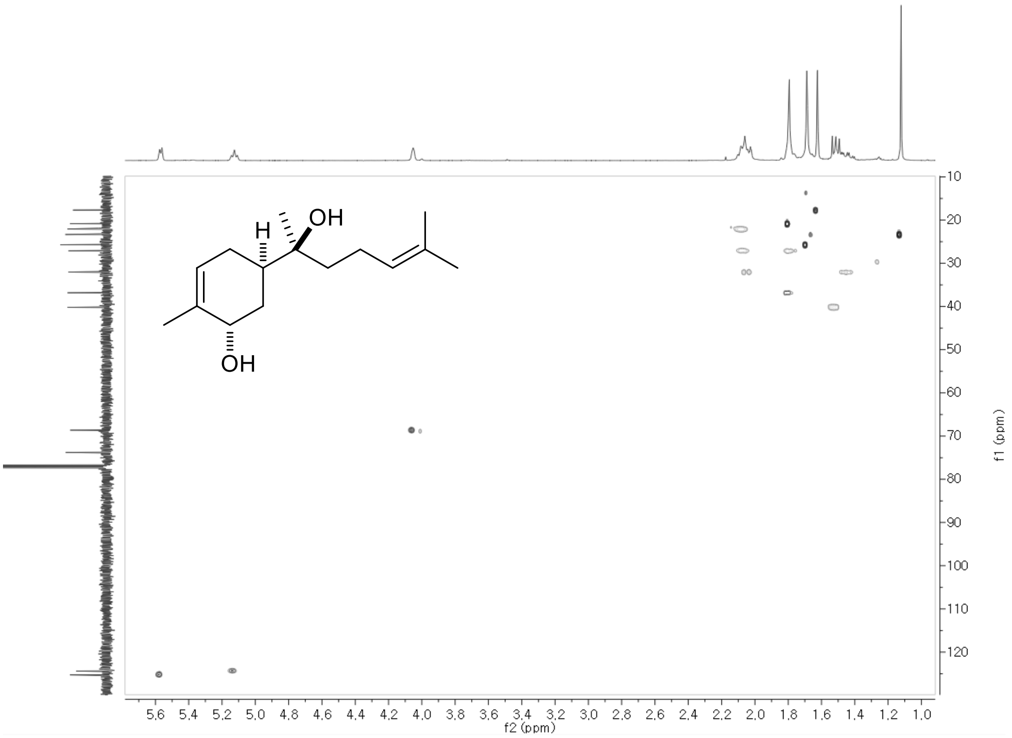

Figure S15. HSQC spectrum of compound **3** recorded at 500 MHz in $CDCl_3$.

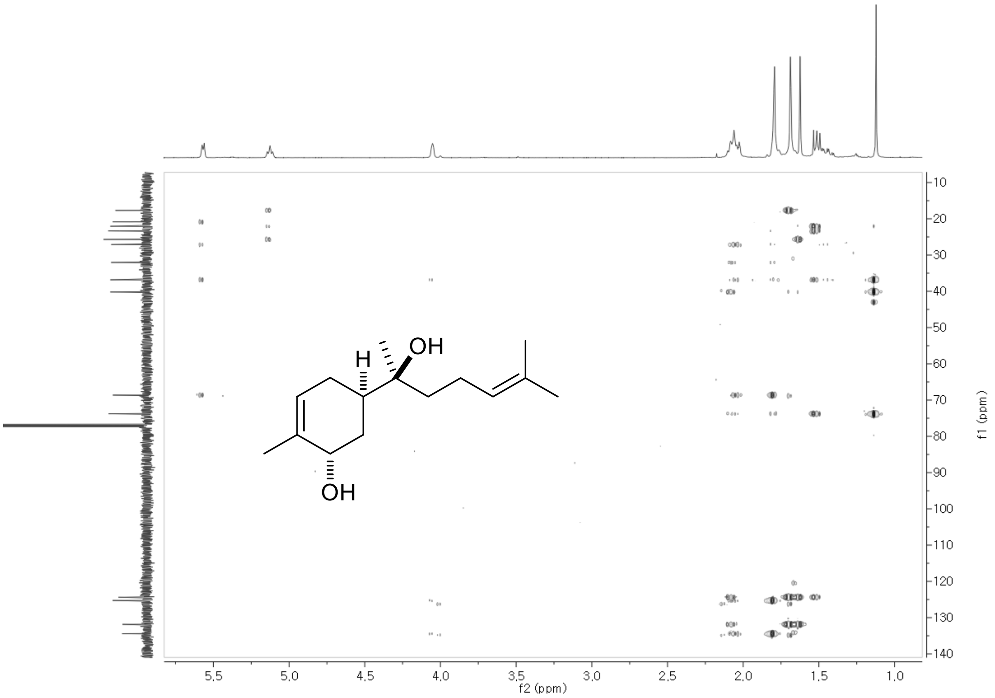

Figure S16. HMBC spectrum of compound **3** recorded at 500 MHz in $CDCl_3$.

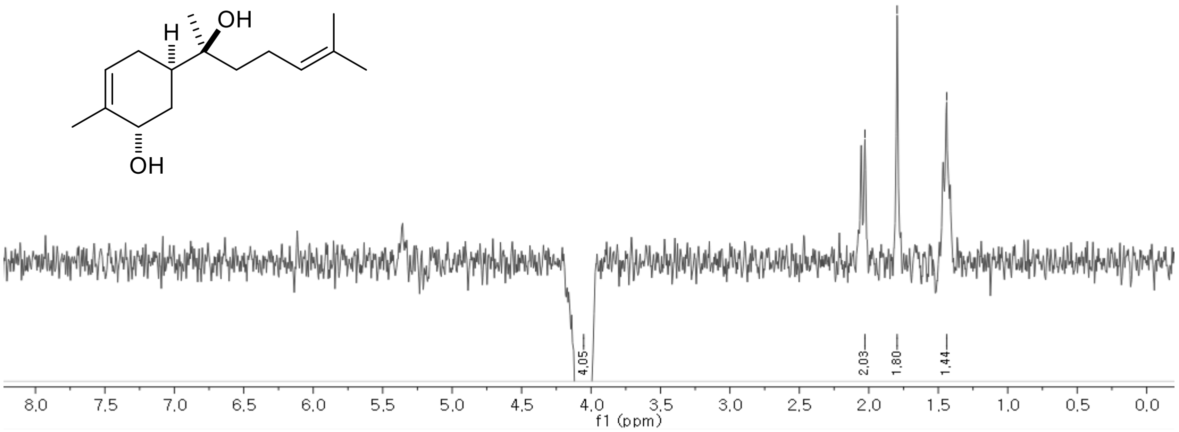

Figure S17. NOE spectrum of compound **3** recorded at 500 MHz in $CDCl_3$.

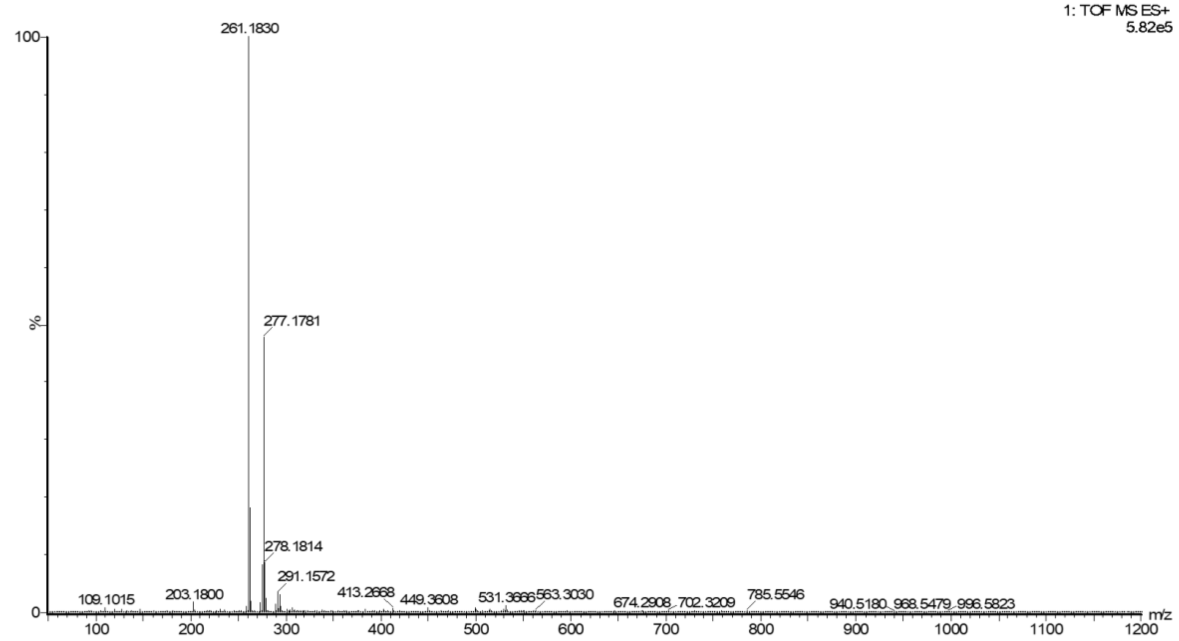

Figure S18. The HRESIMS spectroscopic data of compound **3.**

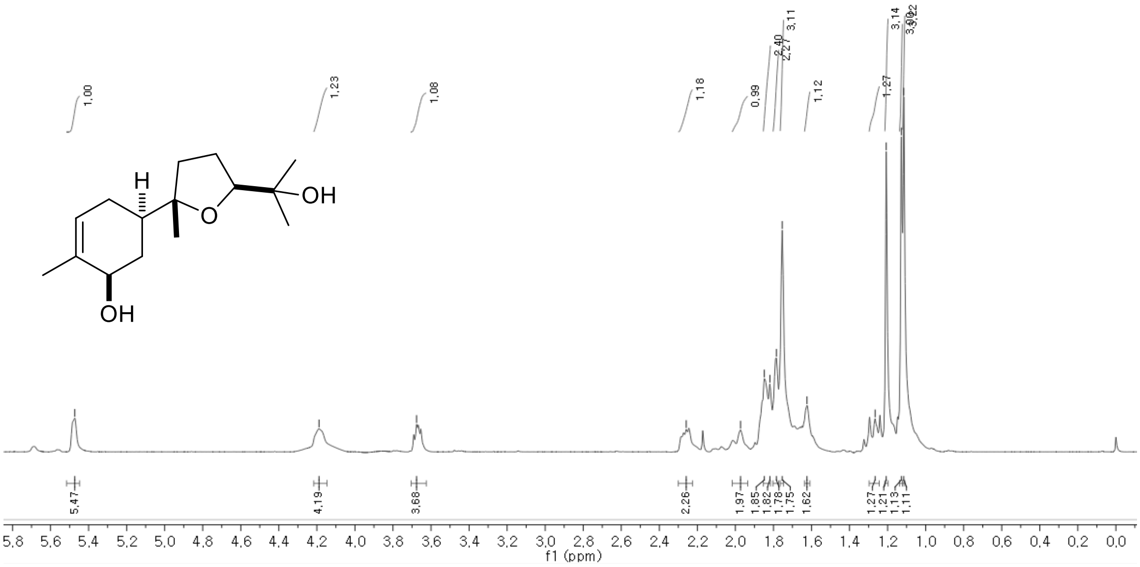

Figure S19. ^{1}H NMR spectrum of compound **4** recorded at 400 MHz in $CDCl_3$.

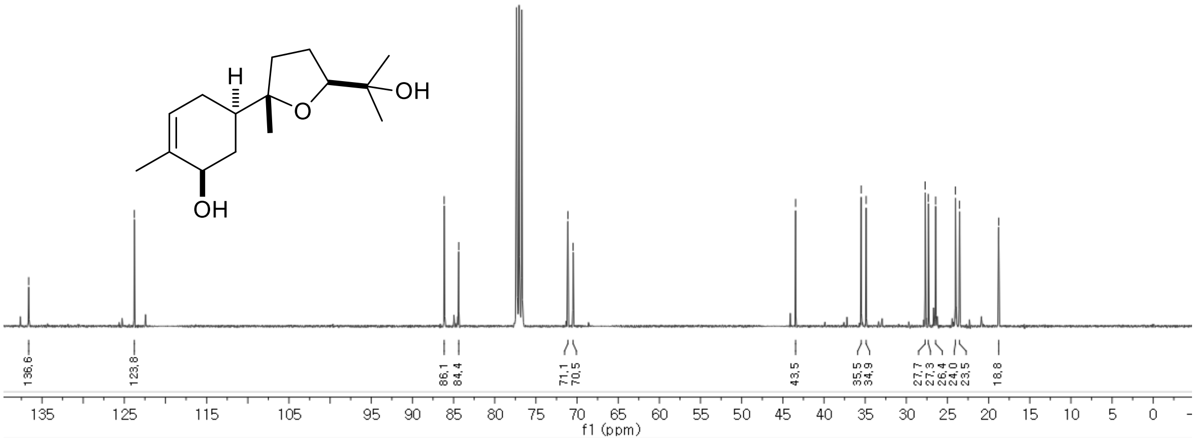

Figure S20. ^{13}C NMR spectrum of compound **4** recorded at 100 MHz in $CDCl_3$.

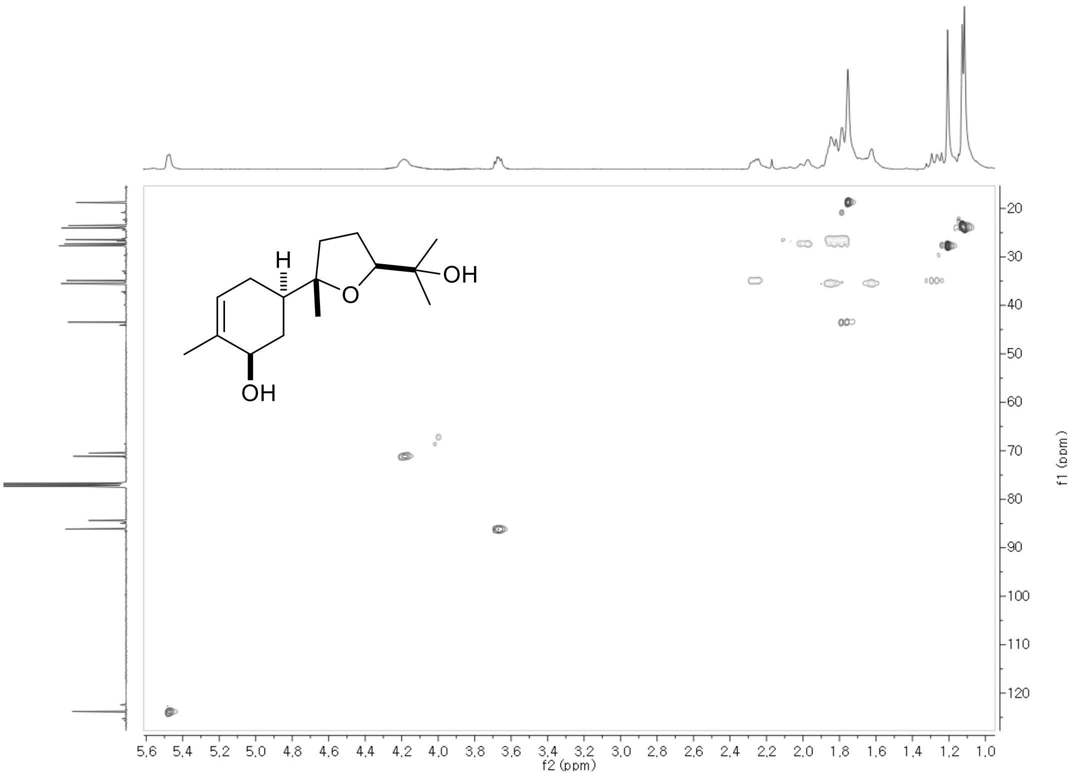

Figure S21. HSQC spectrum of compound **4** recorded at 400 MHz in $CDCl_3$.

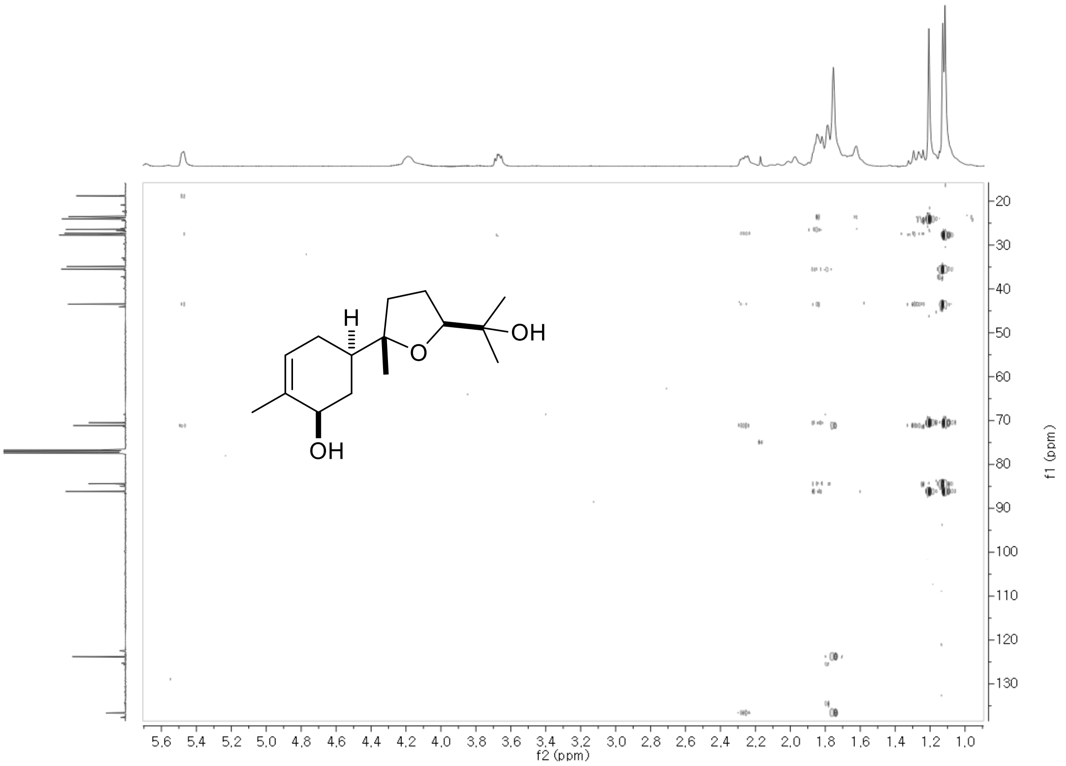

Figure S22. HMBC spectrum of compound **4** recorded at 400 MHz in $CDCl_3$.

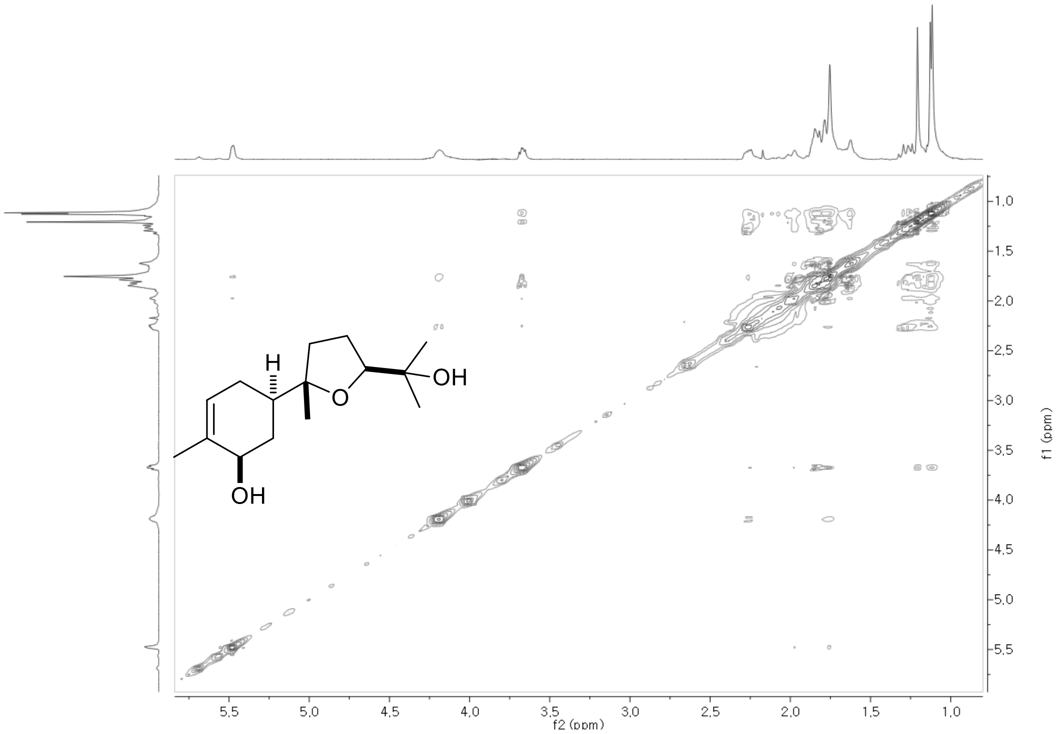

Figure S23. NOESY spectrum of compound **4** recorded at 400 MHz in $CDCl_3$.

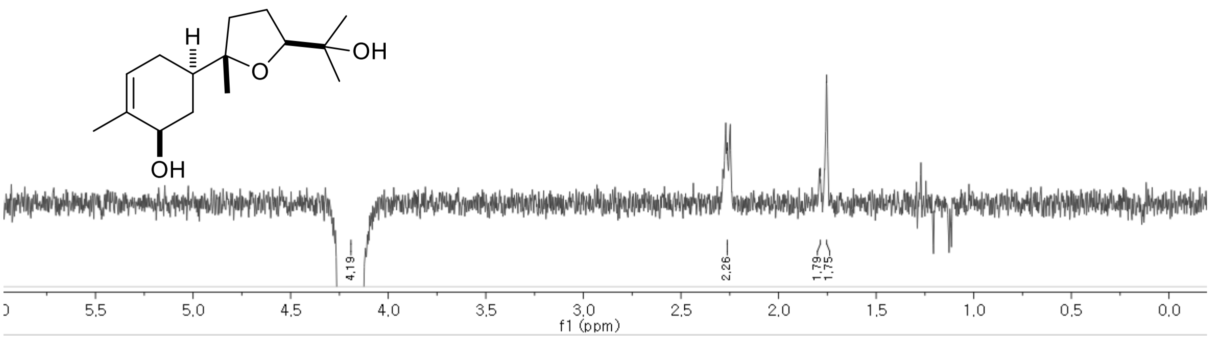

Figure S24. 1D ROESY spectrum of compound **4** recorded at 500 MHz in $CDCl_3$.

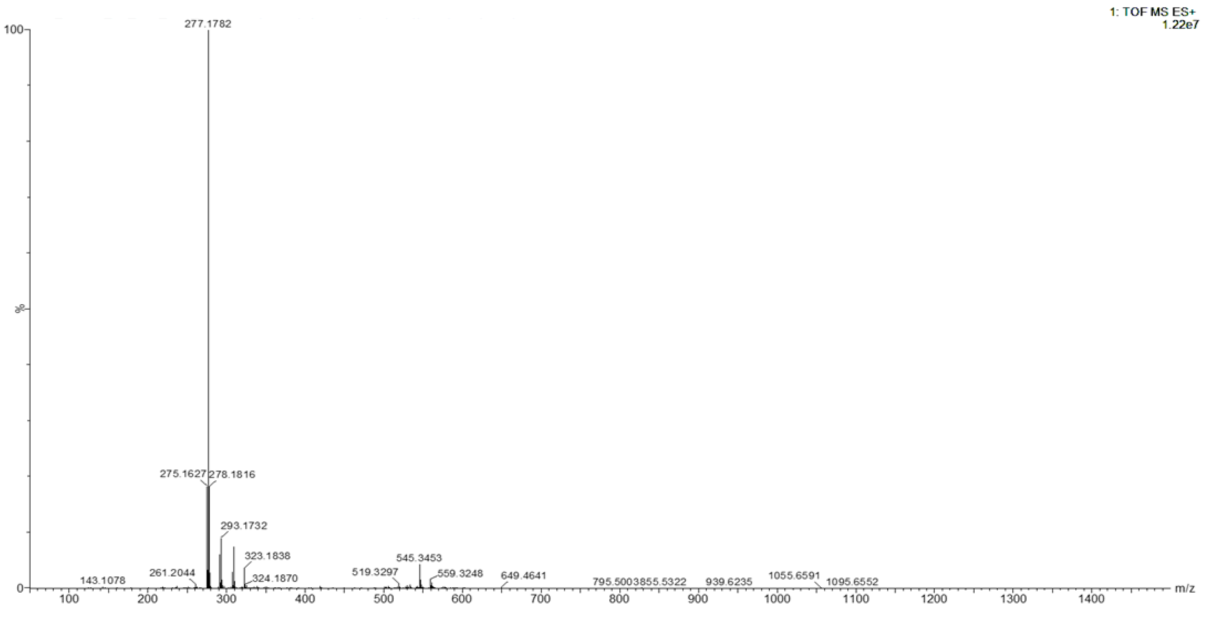

Figure S25. The HRESIMS spectroscopic data of compound **4.**

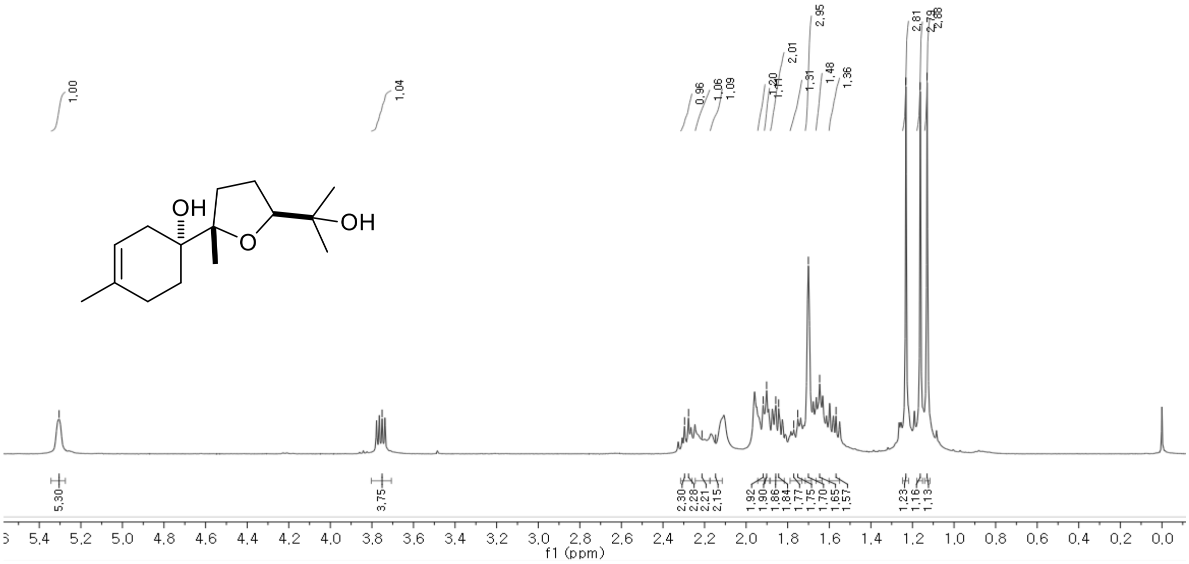

Figure S26. ^{1}H NMR spectrum of compound **5** recorded at 400 MHz in $CDCl_3$.

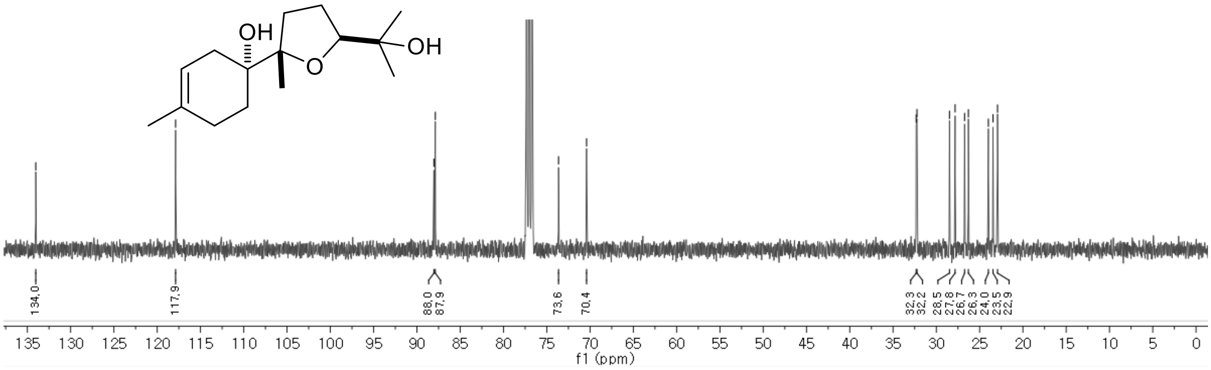

Figure S27. ^{13}C NMR spectrum of compound **5** recorded at 100 MHz in $CDCl_3$.

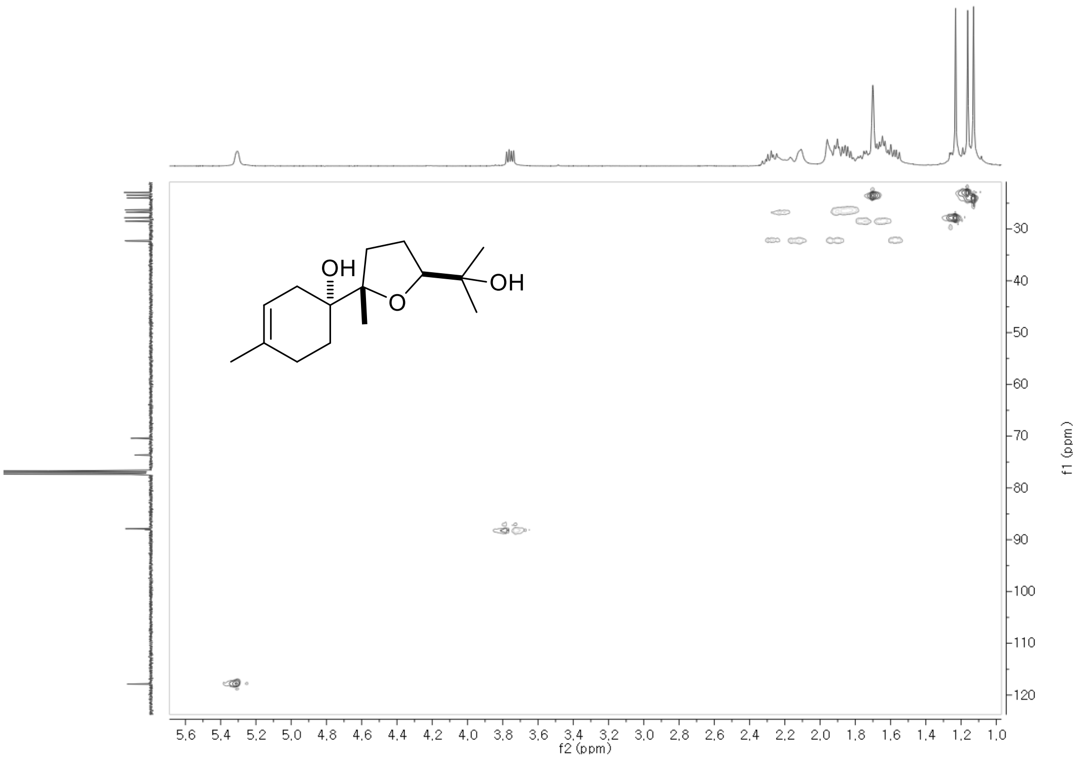

Figure S28. HSQC spectrum of compound **5** recorded at 400 MHz in CDCl3.

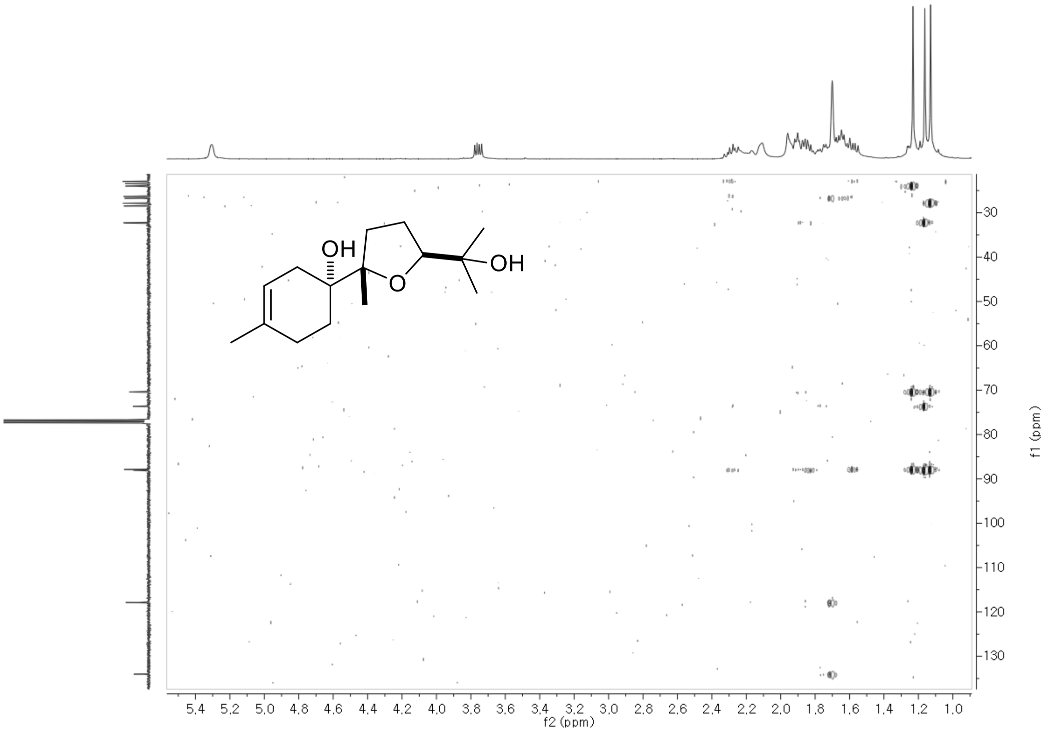

Figure S29. HMBC spectrum of compound **5** recorded at 400 MHz in CDCl3.

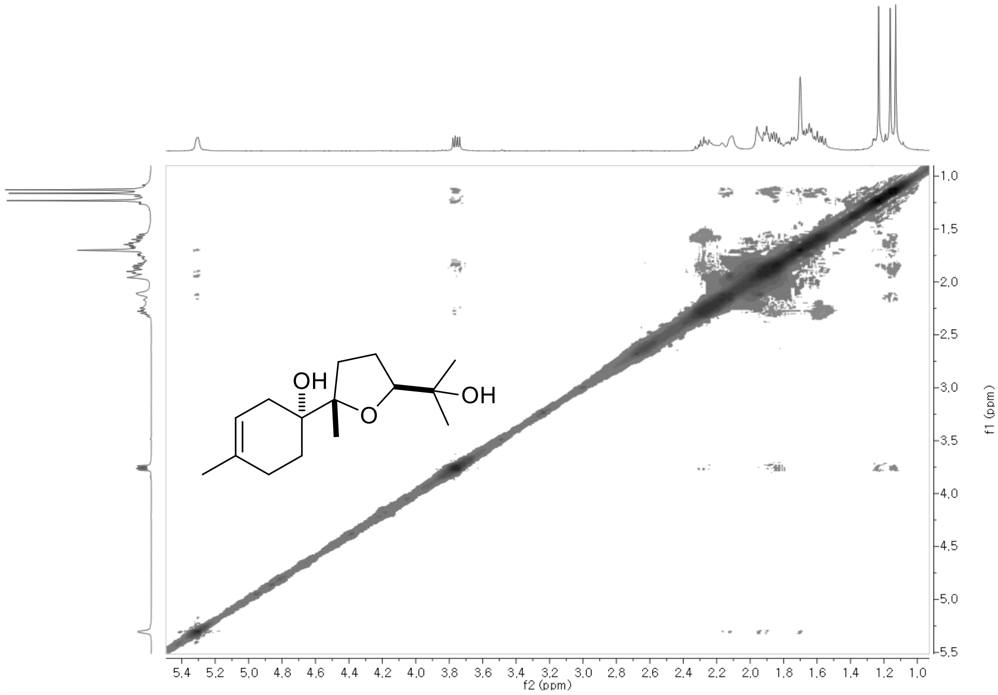

Figure S30. NOESY spectrum of compound **5** recorded at 400 MHz in $CDCl_3$.

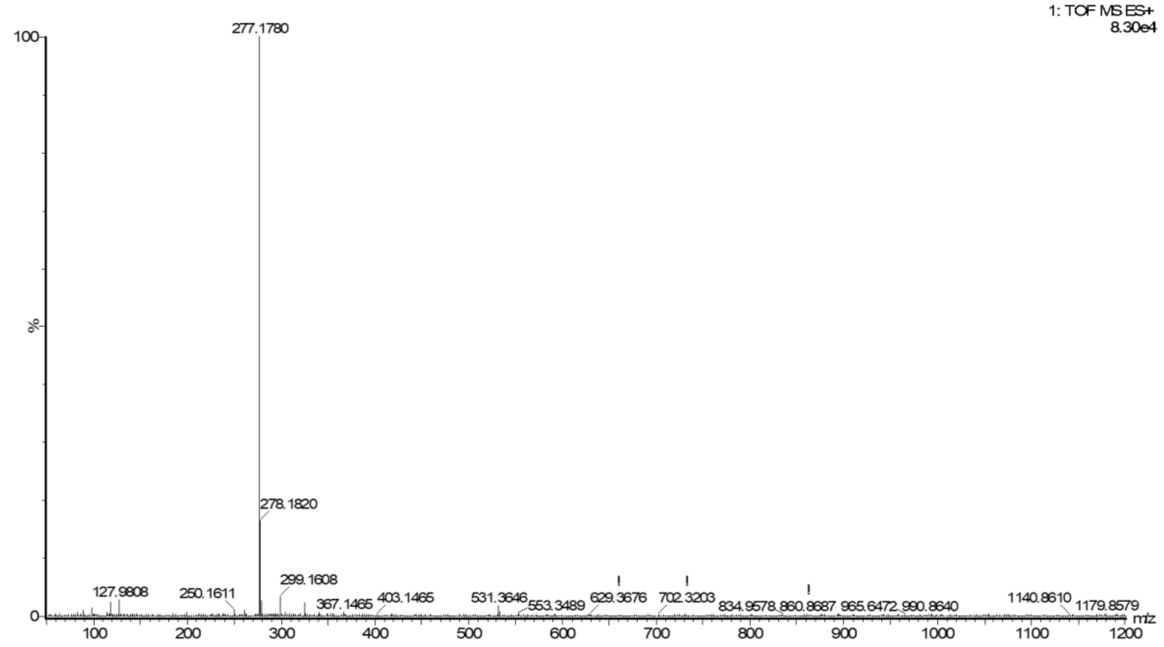

Figure S31. The HRESIMS spectrum of compound **5**.

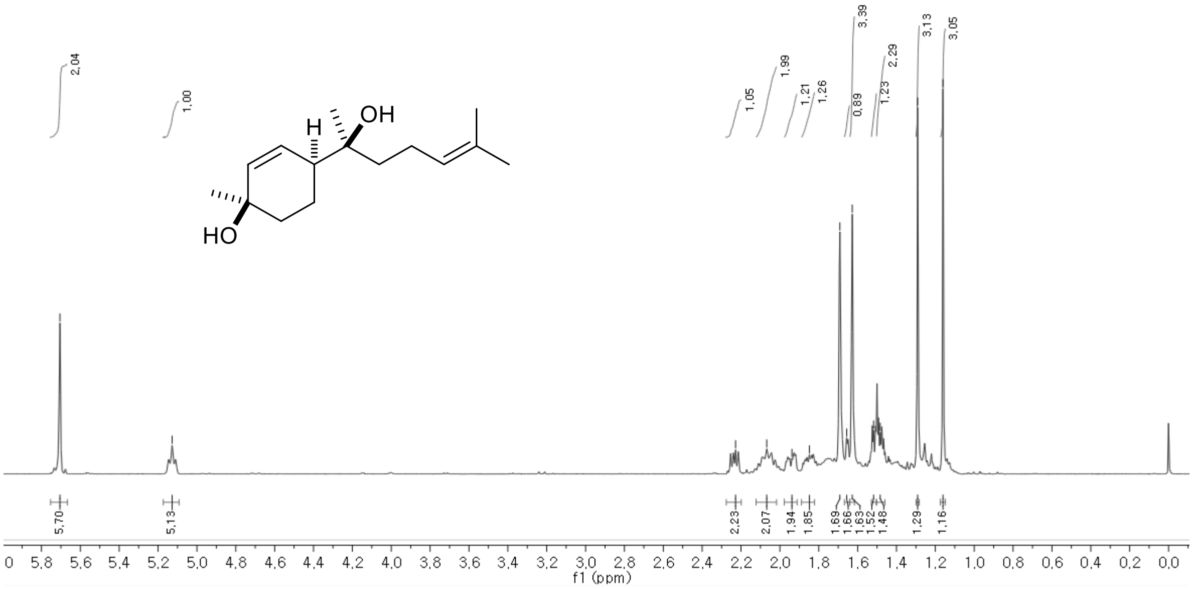

Figure S32. ^{1}H NMR spectrum of compound **6** recorded at 400 MHz in $CDCl_3$.

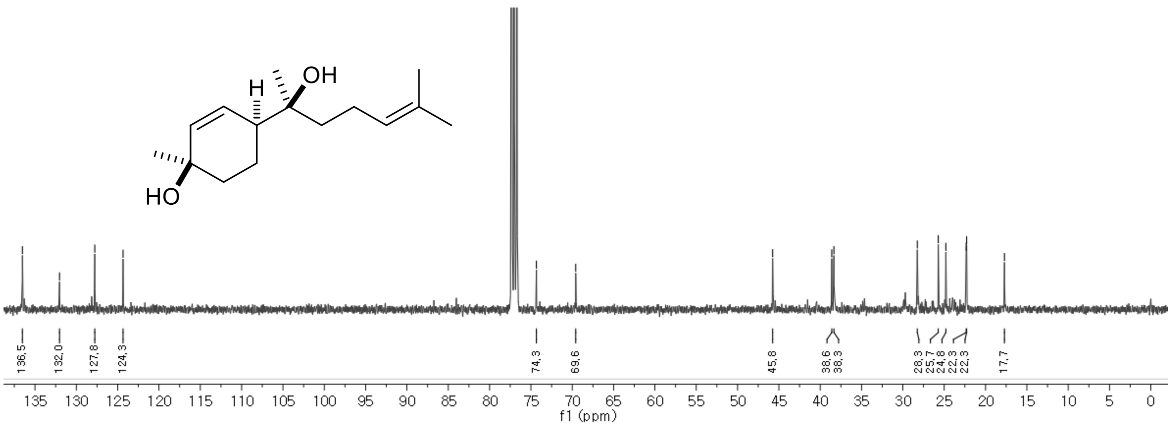

Figure S33. ^{13}C NMR spectrum of compound **6** recorded at 100 MHz in $CDCl_3$.

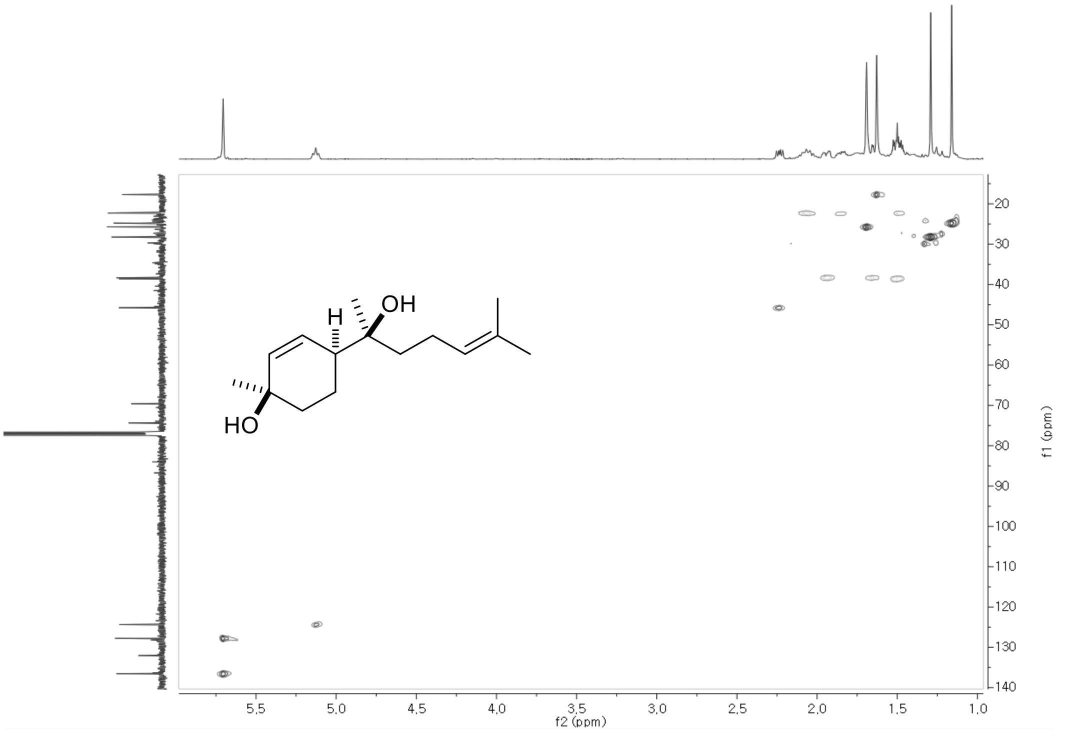

Figure S34. HSQC spectrum of compound **6** recorded at 400 MHz in $CDCl_3$.

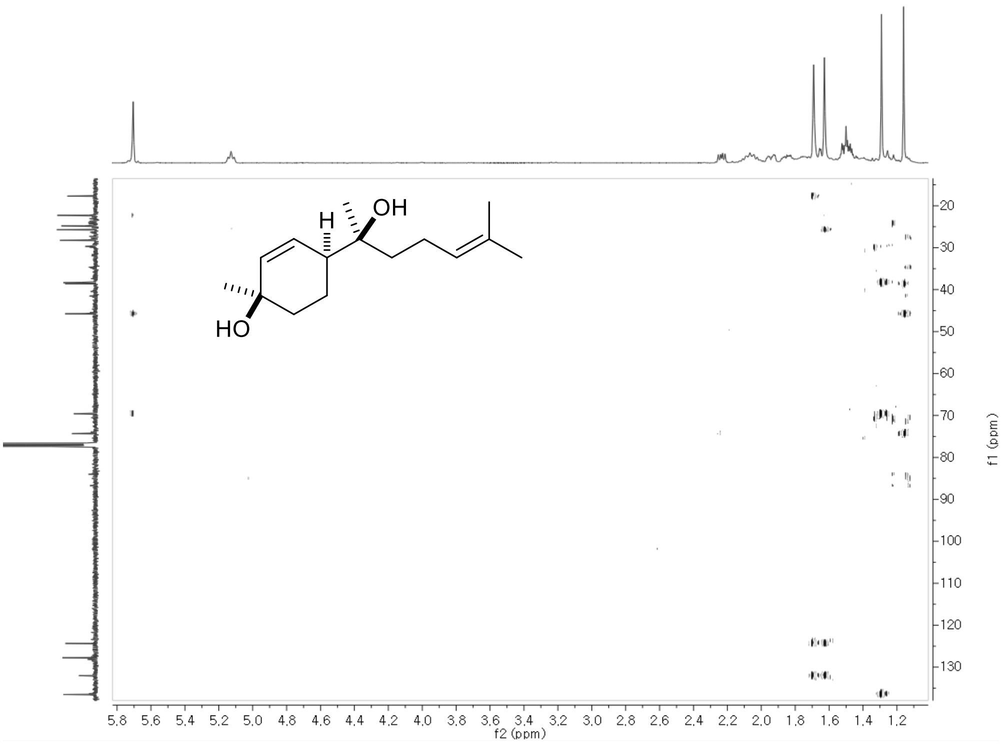

Figure S35. HMBC spectrum of compound **6** recorded at 400 MHz in $CDCl_3$.

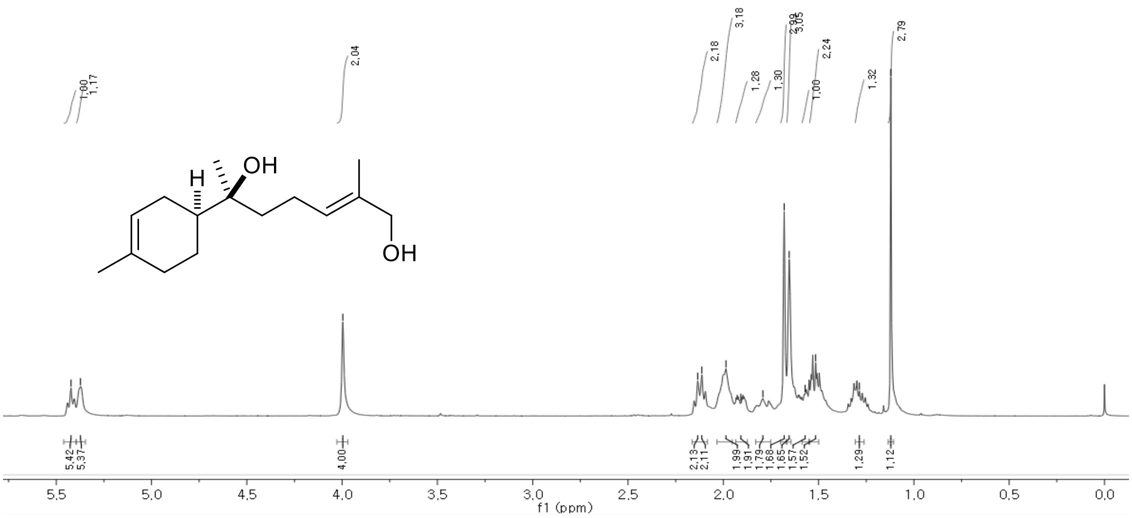

Figure S36. ^1H NMR spectrum of compound **7** recorded at 400 MHz in $CDCl_3$.

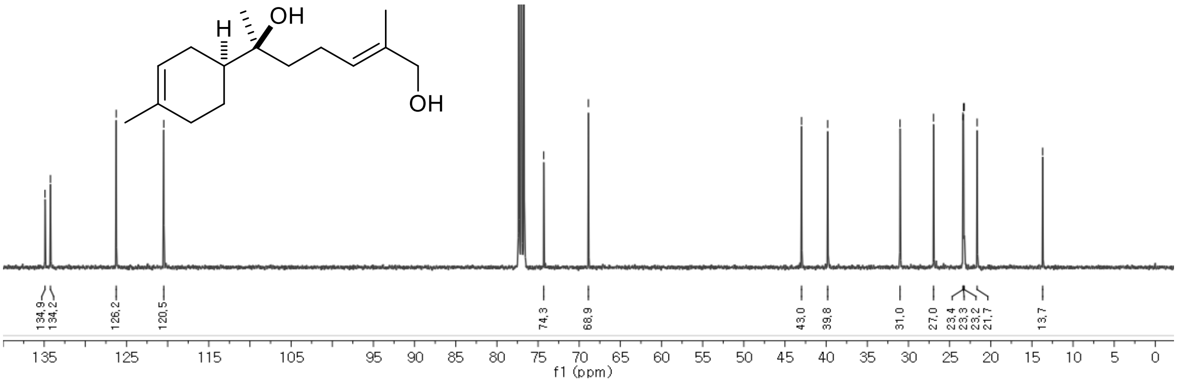

Figure S37. ^{13}C NMR spectrum of compound **7** recorded at 100 MHz in $CDCl_3$.

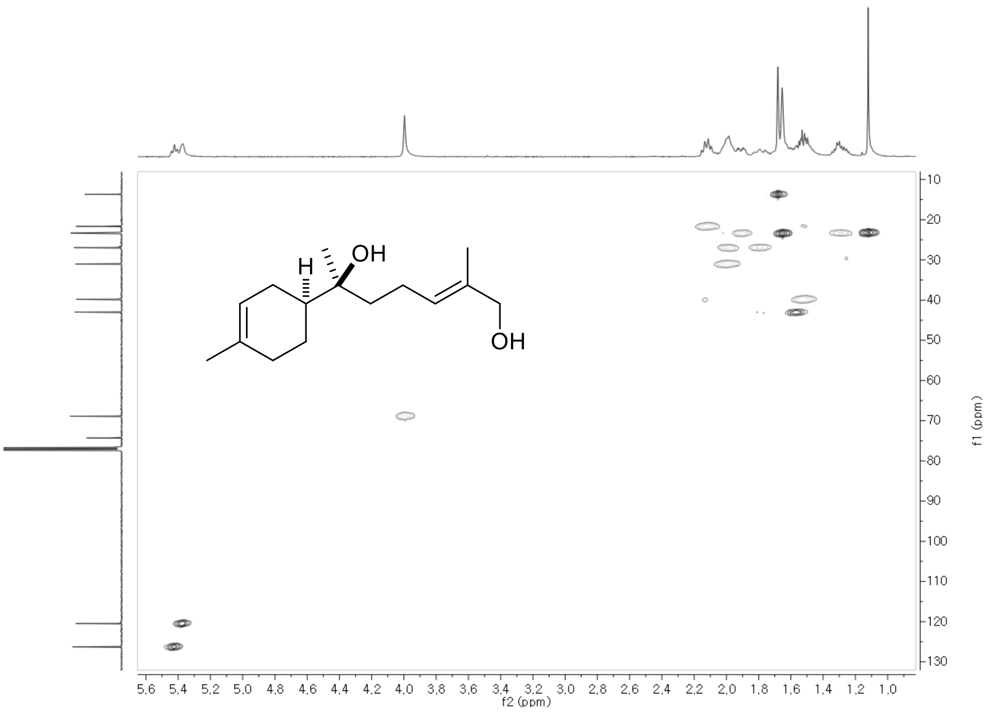

Figure S38. HSQC spectrum of compound **7** recorded at 400 MHz in $CDCl_3$.

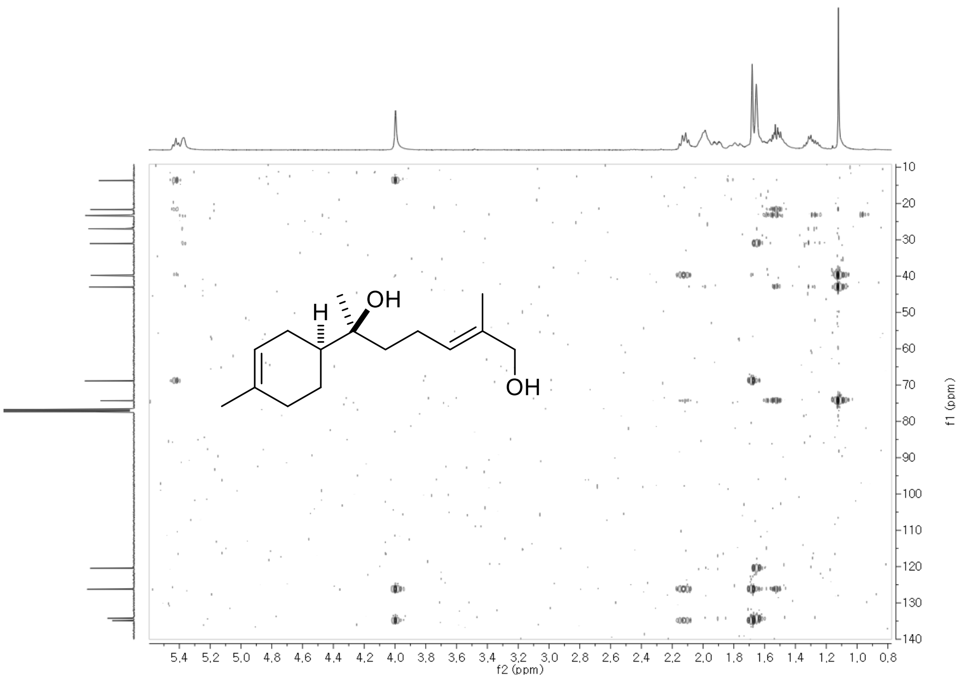

Figure S39. HMBC spectrum of compound **7** recorded at 400 MHz in $CDCl_3$.

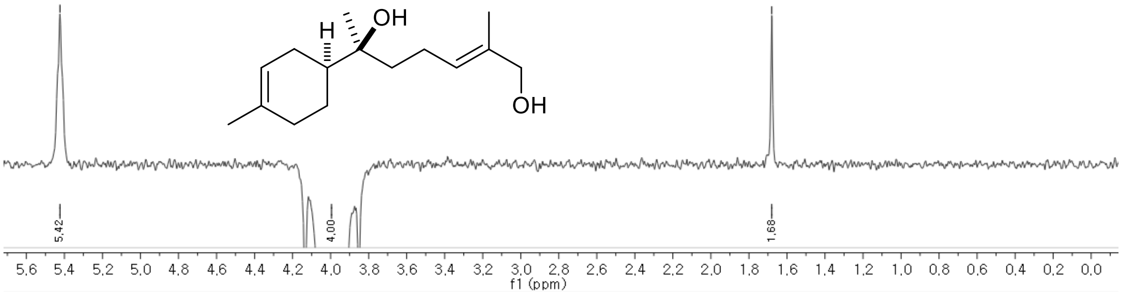

Figure S40. NOE spectrum of compound **7** recorded at 500 MHz in $CDCl_3$.

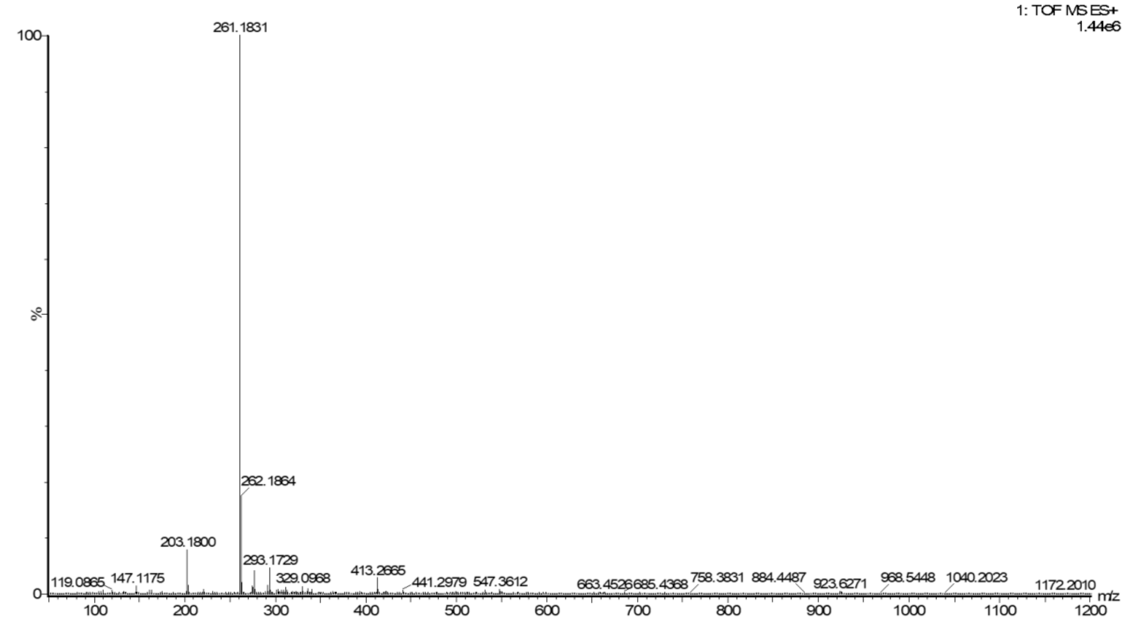

Figure S41. The HRESIMS spectroscopic data of compound **7.**

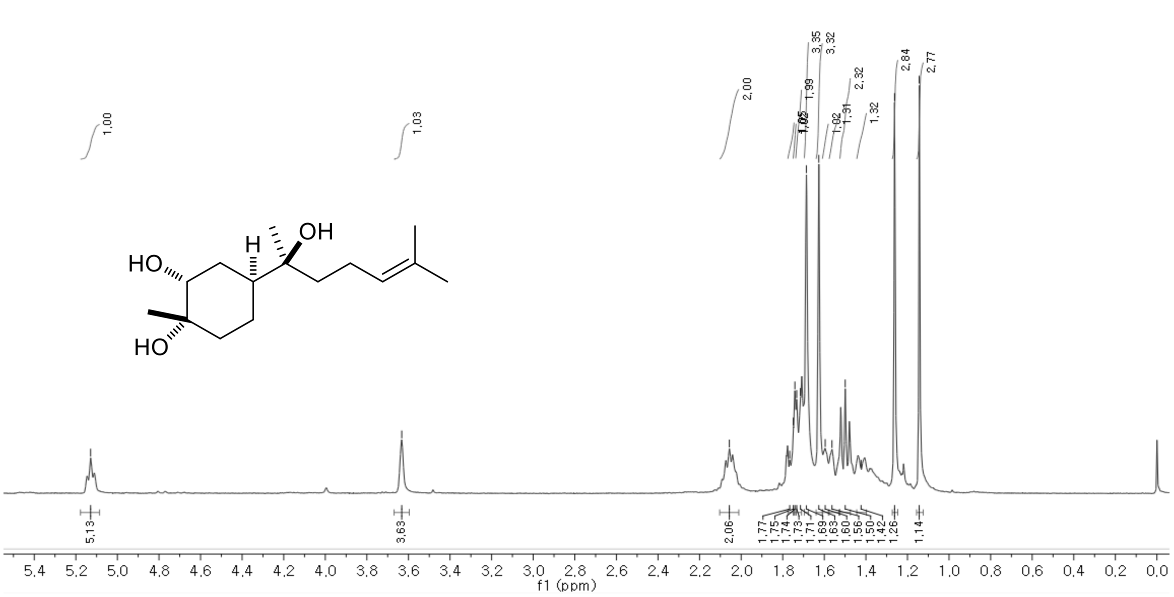

Figure S42. ^{1}H NMR spectrum of compound **8** recorded at 400 MHz in $CDCl_3$.

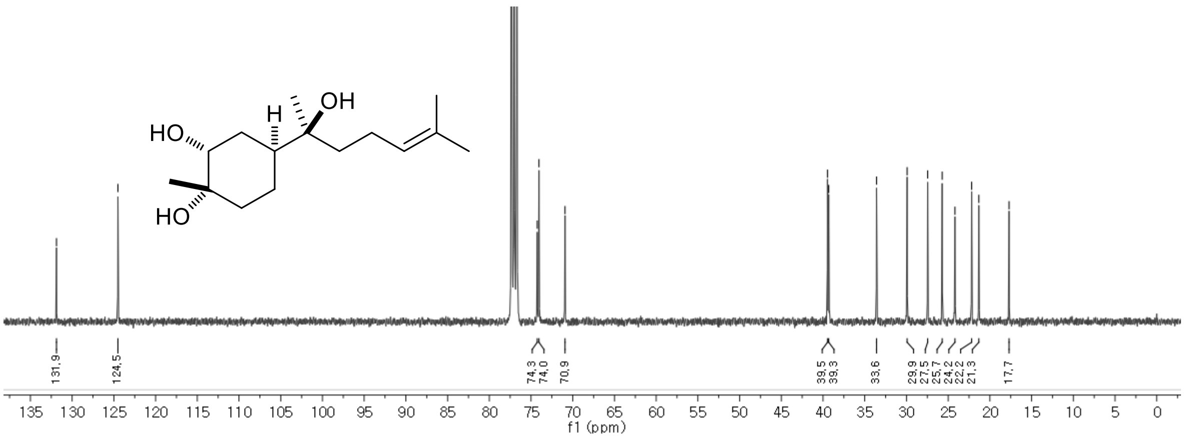

Figure S43. ^{13}C NMR spectrum of compound **8** recorded at 100 MHz in $CDCl_3$.

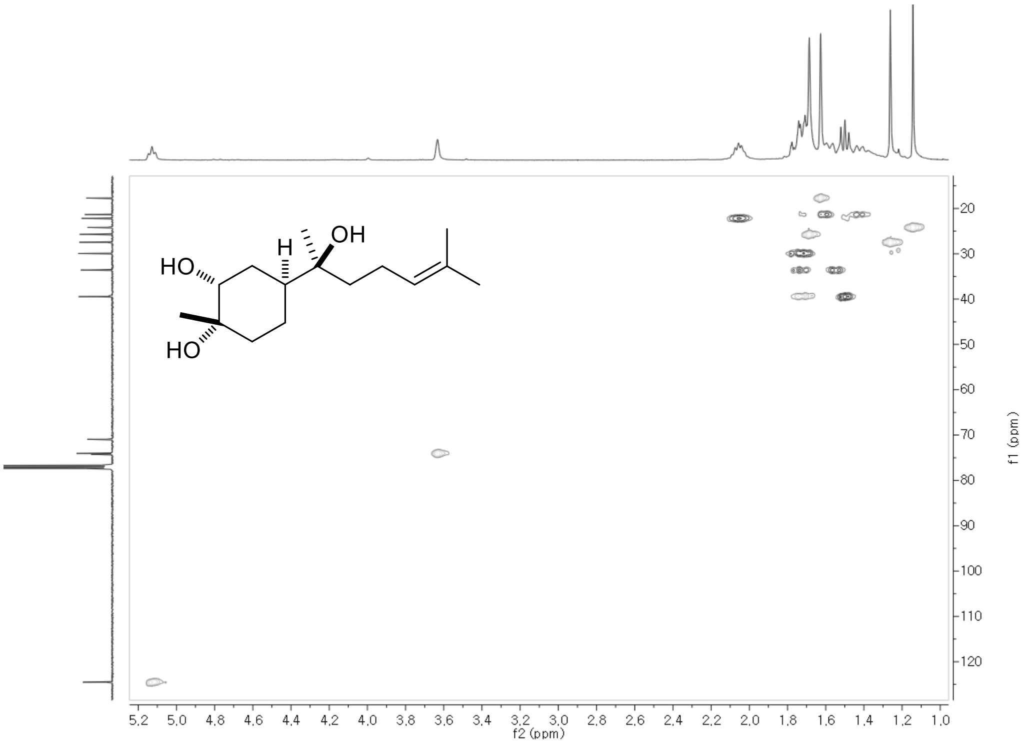

Figure S44. HSQC spectrum of compound **8** recorded at 400 MHz in $CDCl_3$.

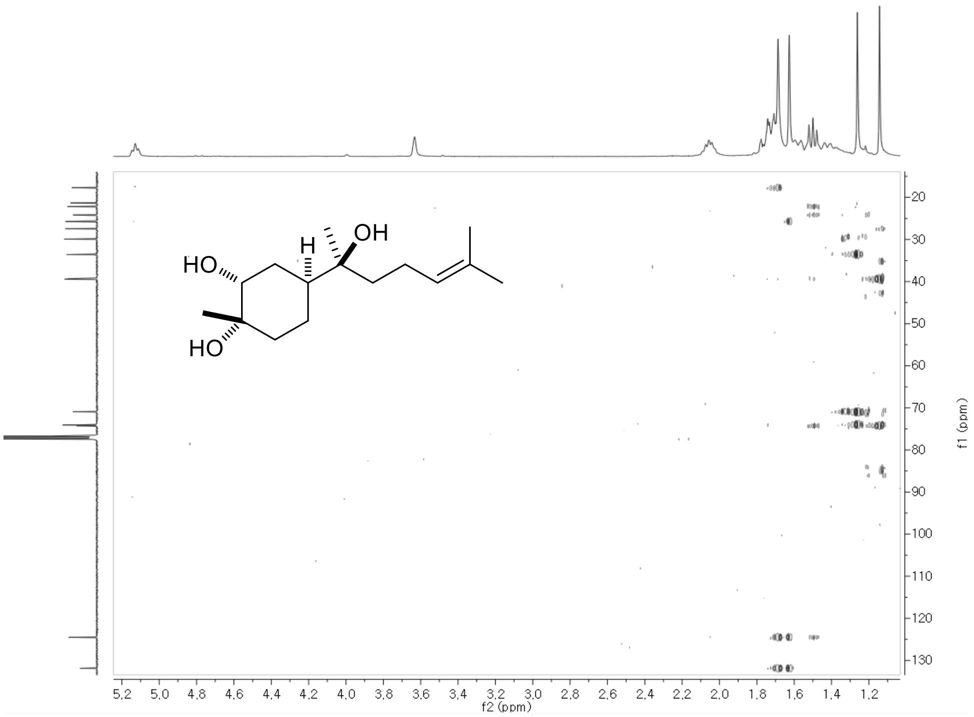

Figure S45. HMBC spectrum of compound **8** recorded at 400 MHz in $CDCl_3$.

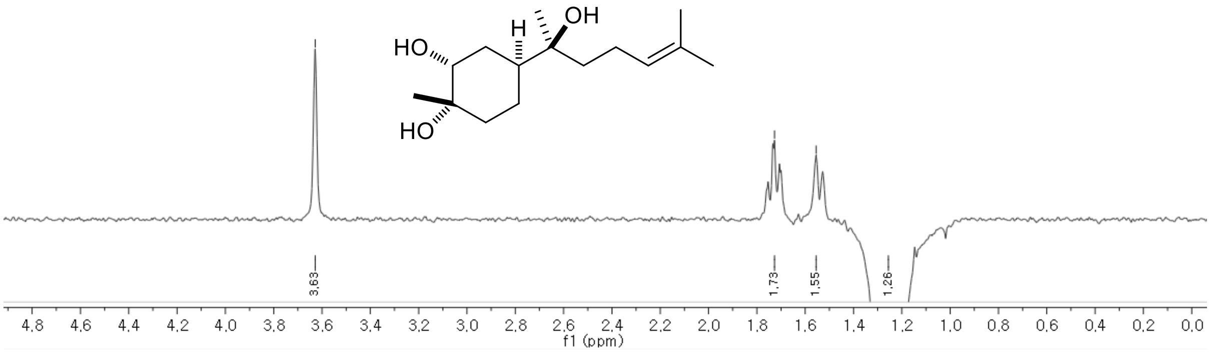

Figure S46. NOE spectrum of compound **8** recorded at 500 MHz in $CDCl_3$.

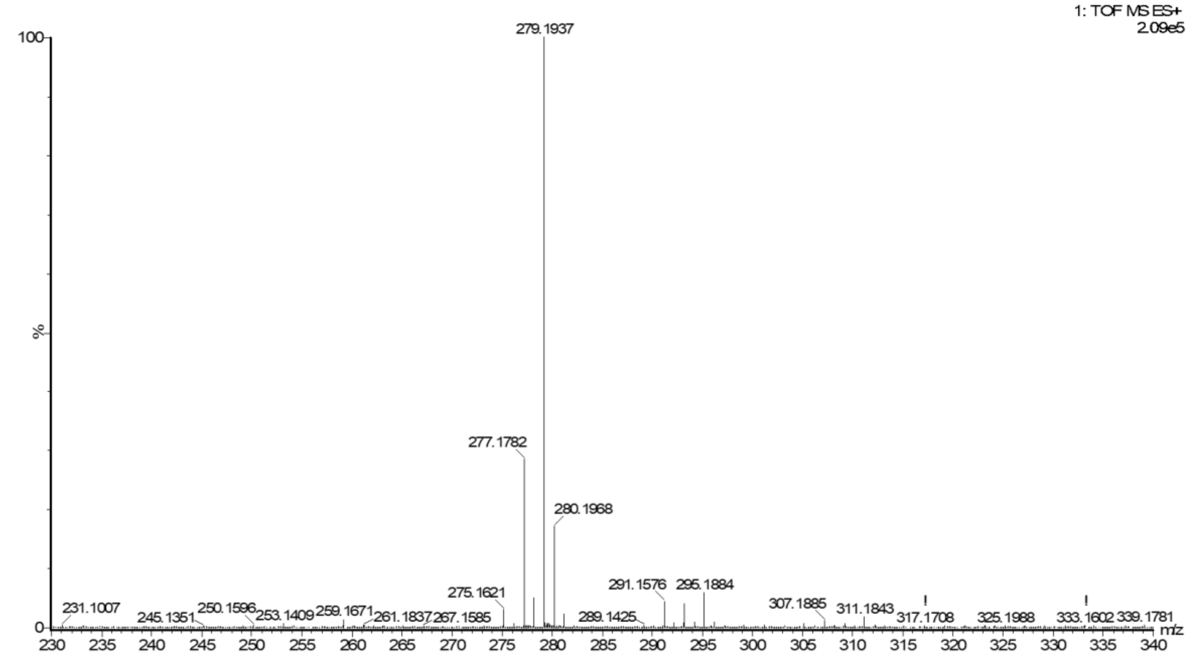

Figure S47. The HRESIMS spectroscopic data of compound **8.**

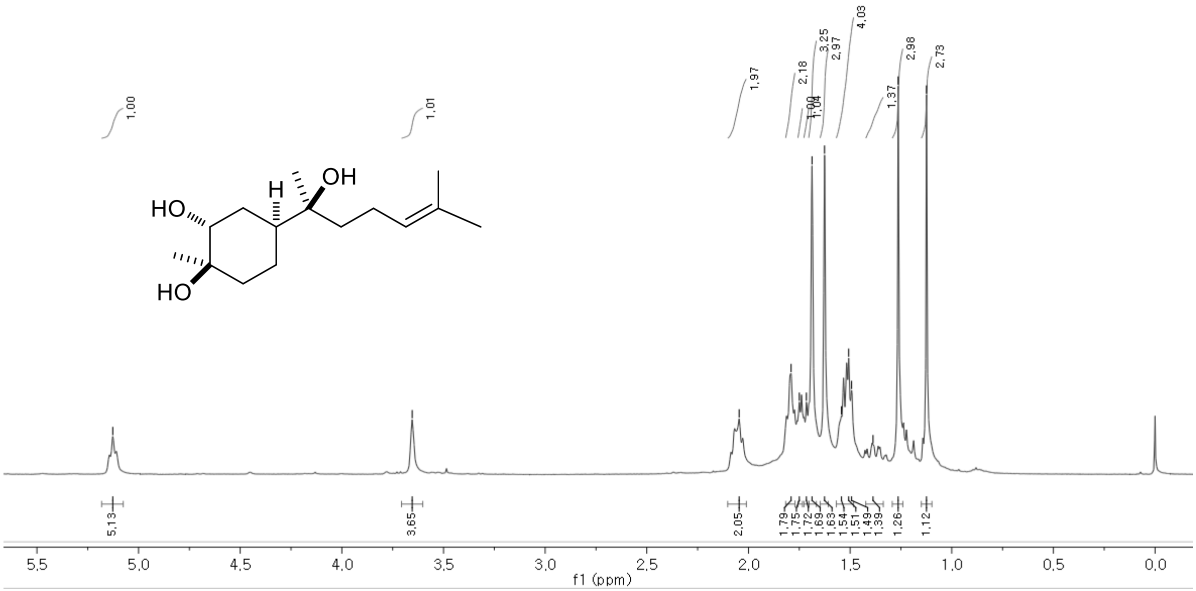

Figure S48. ^{1}H NMR spectrum of compound **9** recorded at 400 MHz in $CDCl_3$.

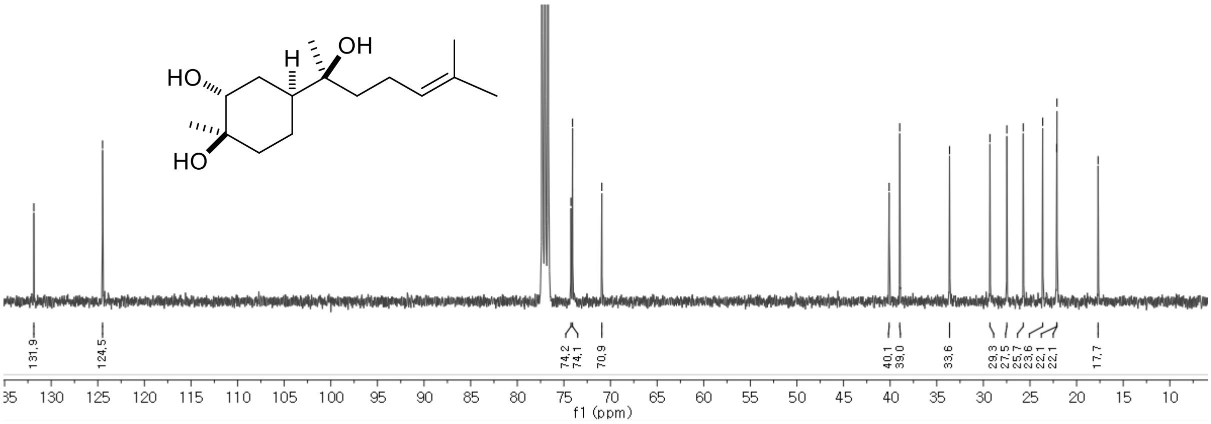

Figure S49. ^{13}C NMR spectrum of compound **9** recorded at 100 MHz in $CDCl_3$.

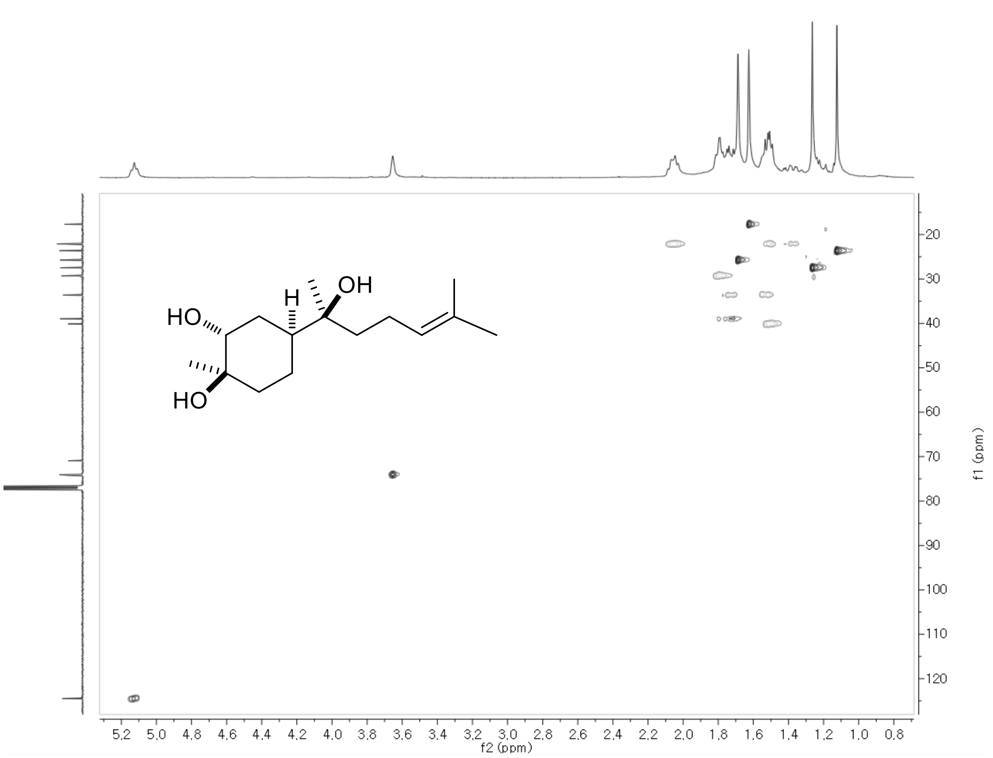

Figure S50. HSQC spectrum of compound **9** recorded at 400 MHz in $CDCl_3$.

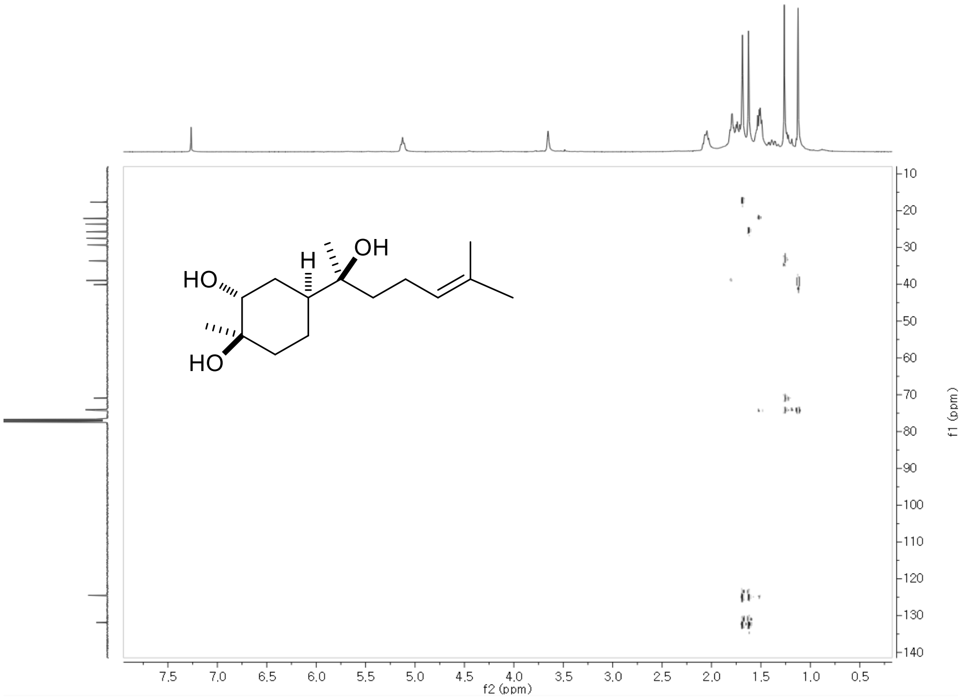

Figure S51. HMBC spectrum of compound **9** recorded at 400 MHz in $CDCl_3$.

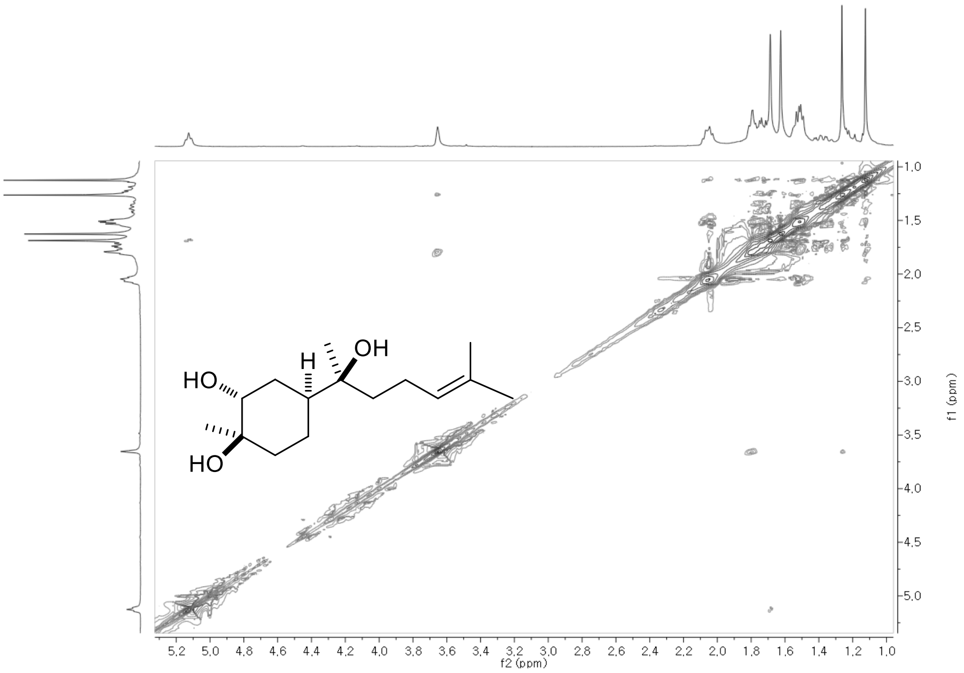

Figure S52. NOESY spectrum of compound **9** recorded at 400 MHz in $CDCl_3$.

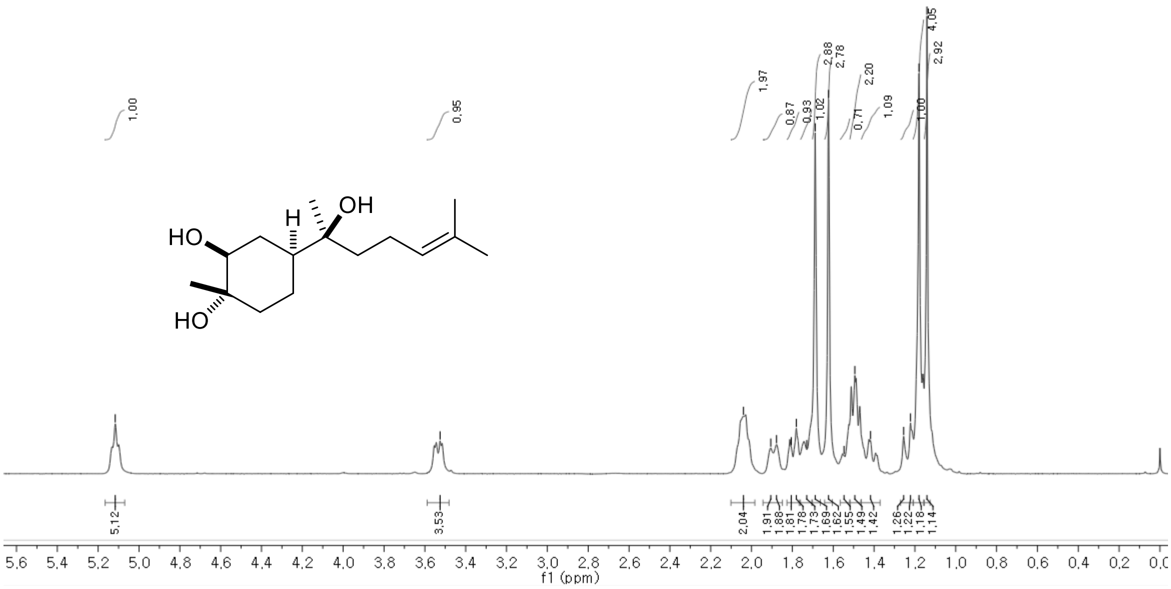

Figure S53. ¹H NMR spectrum of compound **10** recorded at 400 MHz in $CDCl_3$.

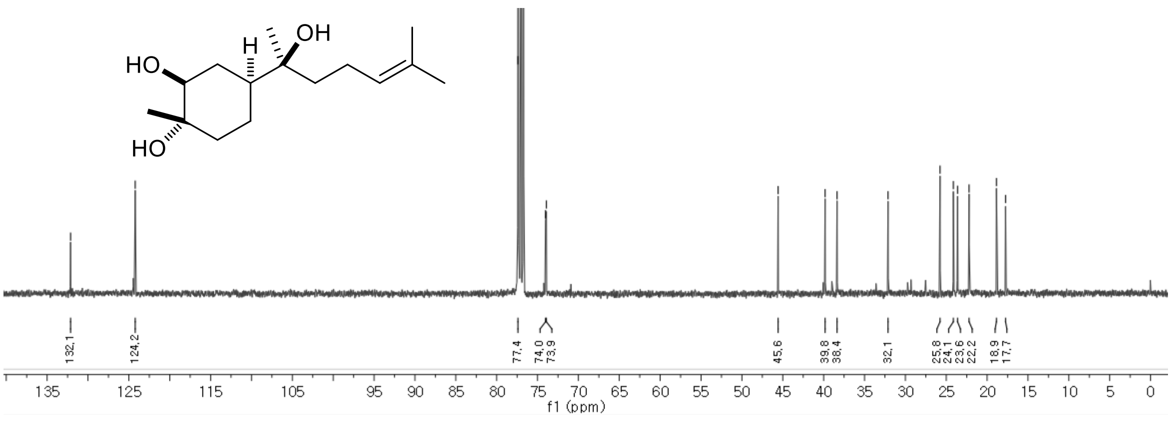

Figure S54. ^{13}C NMR spectrum of compound **10** recorded at 100 MHz in $CDCl_3$.

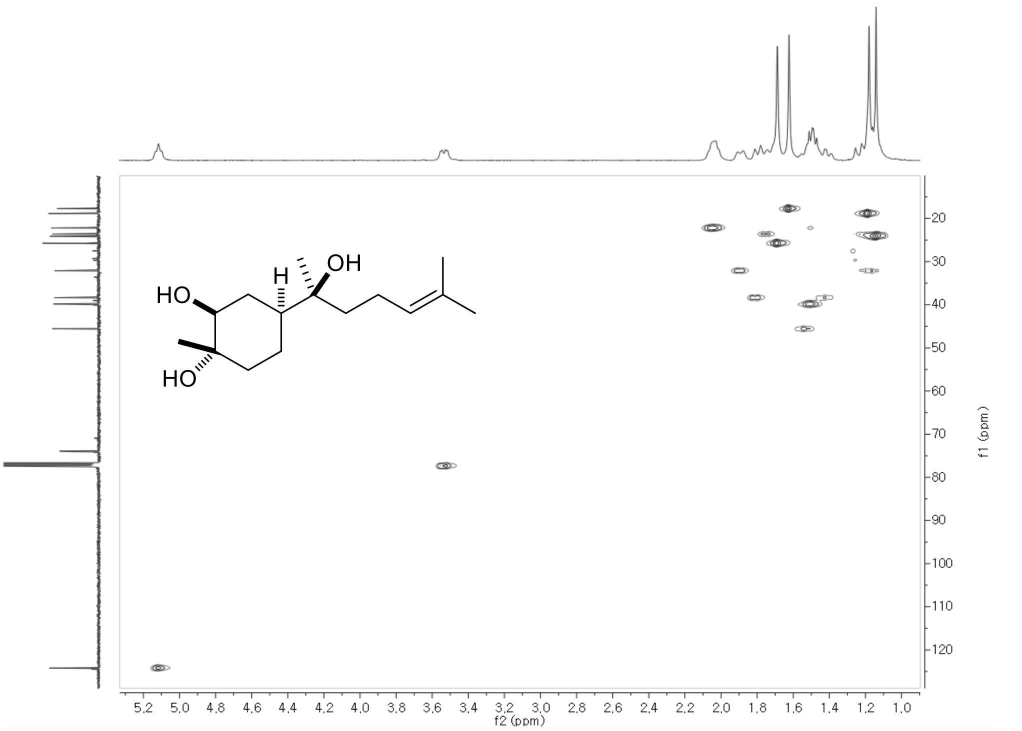

Figure S55. HSQC spectrum of compound **10** recorded at 400 MHz in $CDCl_3$.

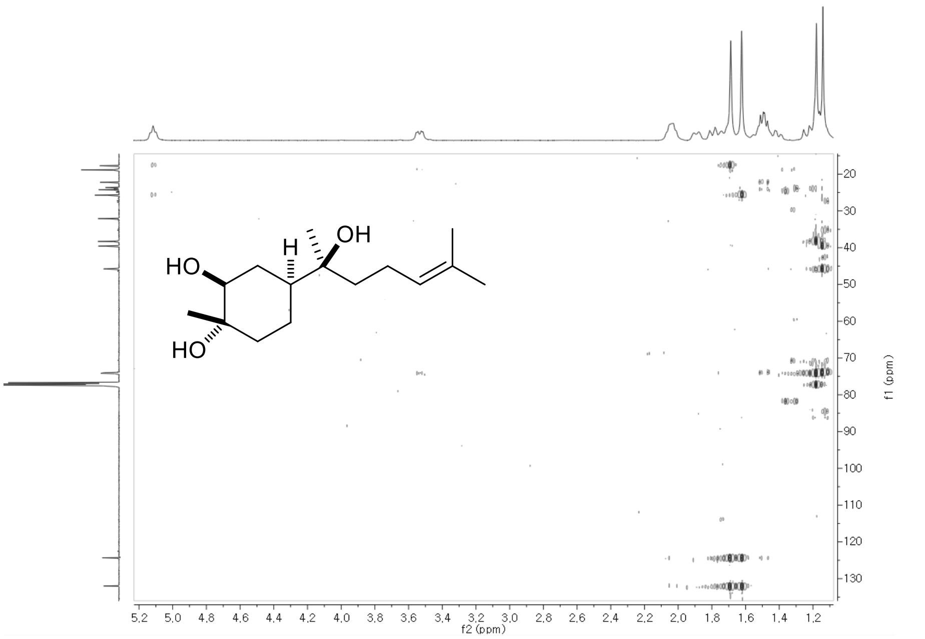

Figure S56. HMBC spectrum of compound **10** recorded at 400 MHz in $CDCl_3$.

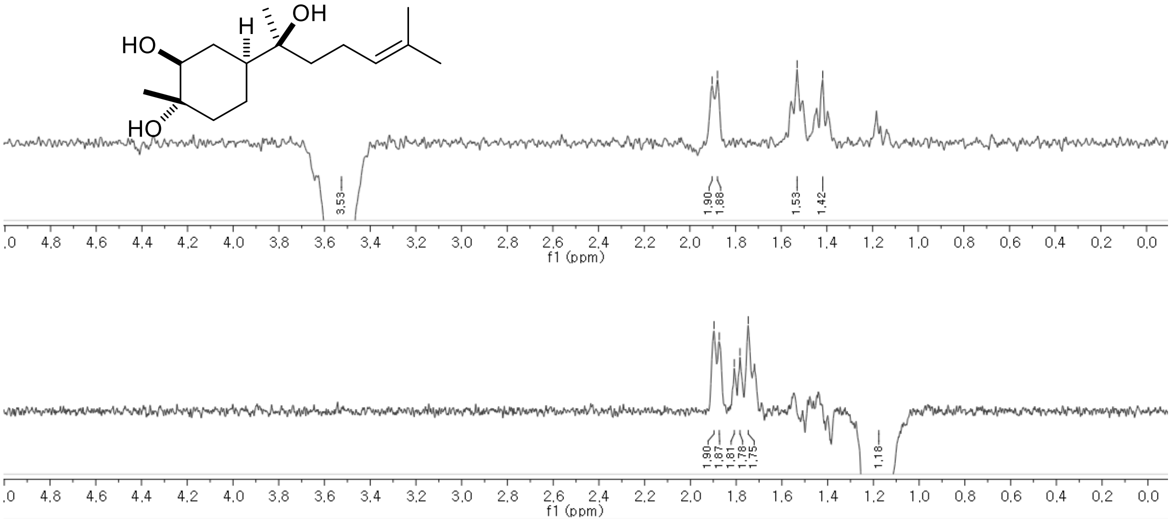

Figure S57. NOE spectrum of compound **10** recorded at 500 MHz in $CDCl_3$.

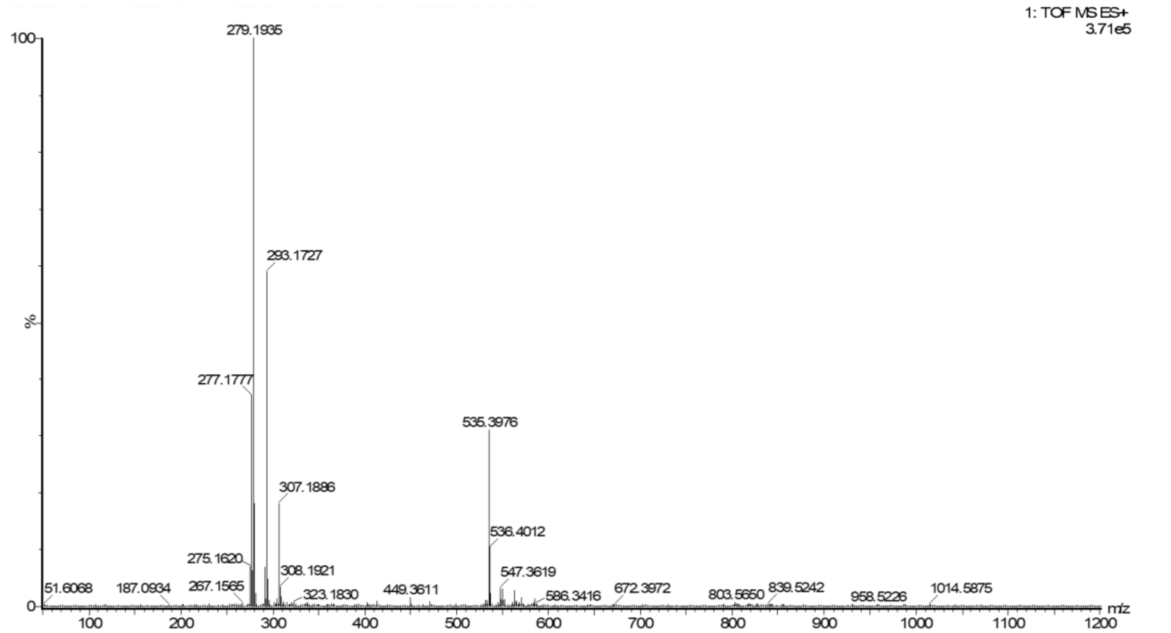

Figure S58. The HRESIMS spectroscopic data of compound **10.**

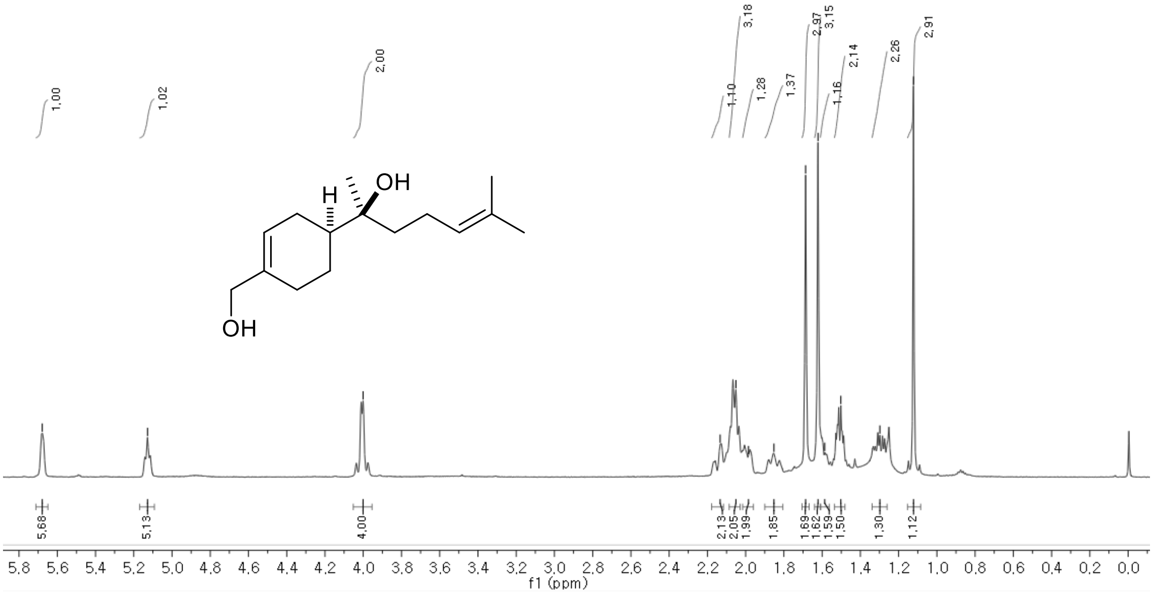

Figure S59. ^{1}H NMR spectrum of compound **11** recorded at 400 MHz in $CDCl_3$.

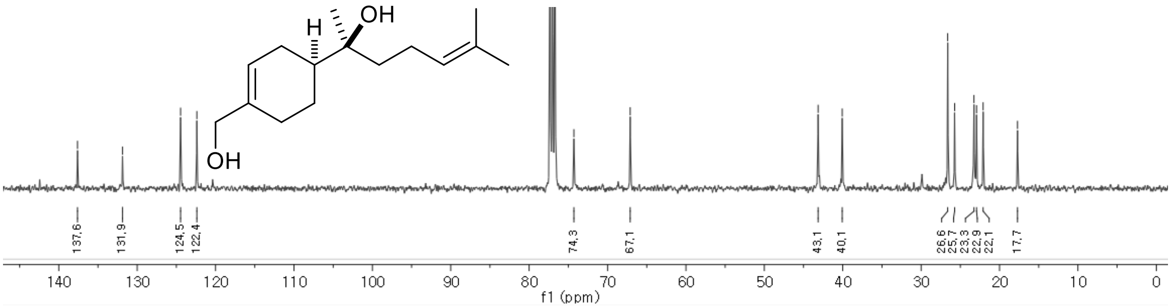

Figure S60. ^{13}C NMR spectrum of compound **11** recorded at 100 MHz in $CDCl_3$.

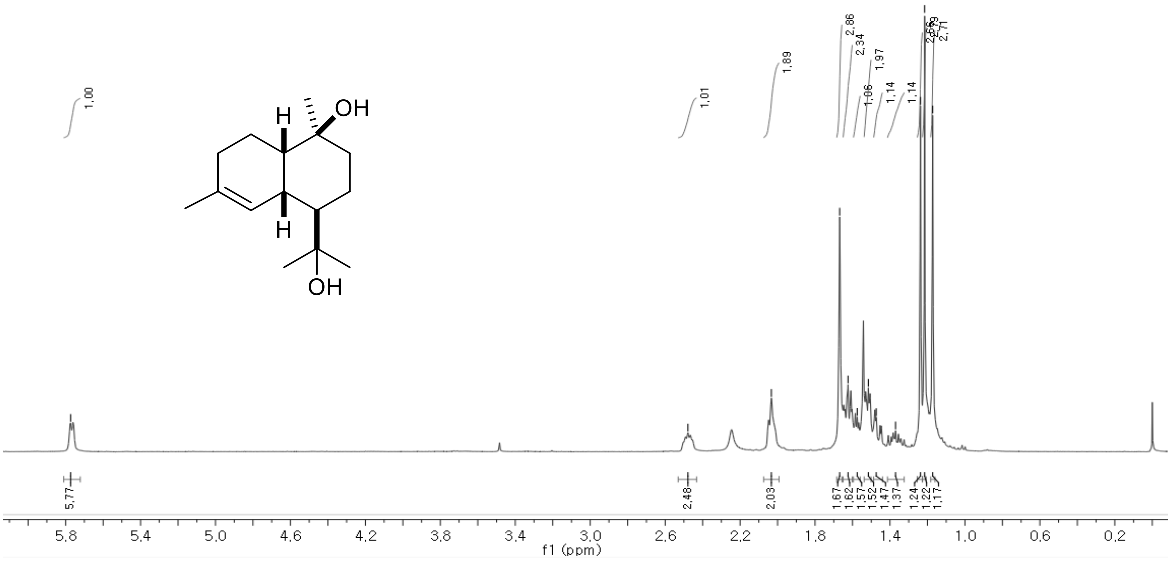

Figure S61. ^{1}H NMR spectrum of compound **12** recorded at 400 MHz in $CDCl_3$.

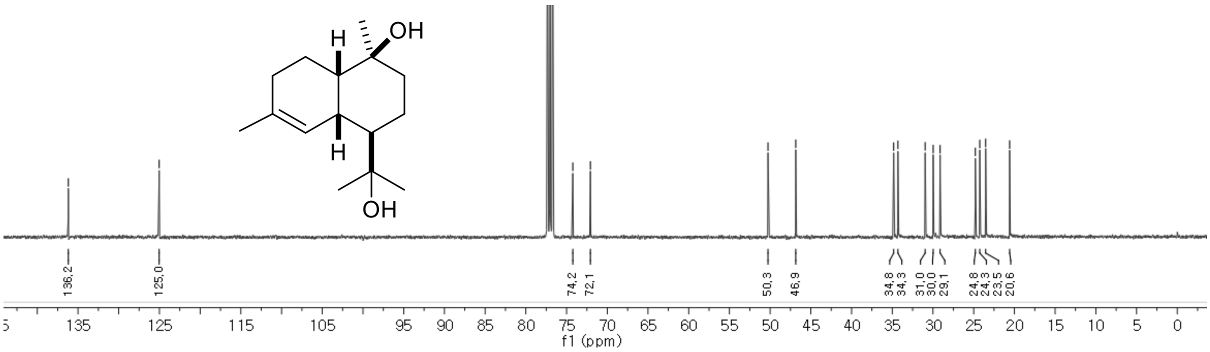

Figure S62. ^{13}C NMR spectrum of compound **12** recorded at 100 MHz in $CDCl_3$.
